# Intrinsically Re-curable Photopolymers Containing Dynamic Thiol-Michael Bonds

**DOI:** 10.1021/jacs.2c03525

**Published:** 2022-06-24

**Authors:** Connor J. Stubbs, Anissa L. Khalfa, Viviane Chiaradia, Joshua C. Worch, Andrew P. Dove

**Affiliations:** School of Chemistry, University of Birmingham, Birmingham B15 2TT, U.K.

## Abstract

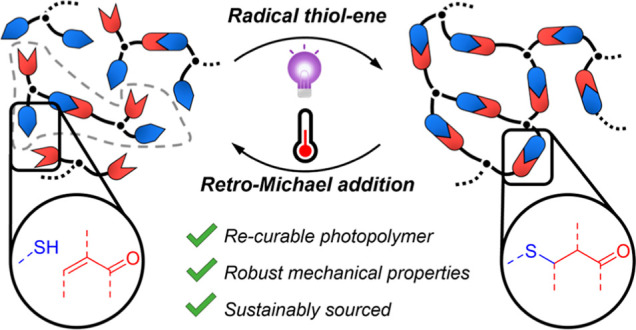

The development of photopolymers that can be depolymerized and subsequently re-cured using the same light stimulus presents a significant technical challenge. A bio-sourced terpenoid structure, l-carvone, inspired the creation of a re-curable photopolymer in which the orthogonal reactivity of an irreversible thioether and a dynamic thiol-Michael bond enables both photopolymerization and thermally driven depolymerization of mechanically robust polymer networks. The di-alkene containing l-carvone was partially reacted with a multi-arm thiol to generate a non-crosslinked telechelic photopolymer. Upon further UV exposure, the photopolymer crosslinked into a mechanically robust network featuring reversible Michael bonds at junction points that could be activated to revert, or depolymerize, the network into a viscous telechelic photopolymer. The regenerated photopolymer displayed intrinsic re-curability over two recycles while maintaining the desirable thermomechanical properties of a conventional network: insolubility, resistance to stress relaxation, and structural integrity up to 170 °C. Our findings present an on-demand, re-curable photopolymer platform based on a sustainable feedstock.

## Introduction

Despite the widespread challenges in recycling polymer networks, they remain a critical material class for use in foams, coatings, and rubbers.^1,2^ The covalent crosslinks, synonymous to polymeric networks, impart the materials with excellent thermal stability and mechanical robustness. These desirable properties, regrettably, hamper conventional recycling techniques and limit reuse once the network is formed. Overcoming these challenges and designing crosslinked materials with in-built recycling has been a key research focus for the past decade. However, the rapidly growing field of photopolymers (liquid formulations that generate polymer networks after a light stimulus) has lacked the same focus.

**Figure 1:**
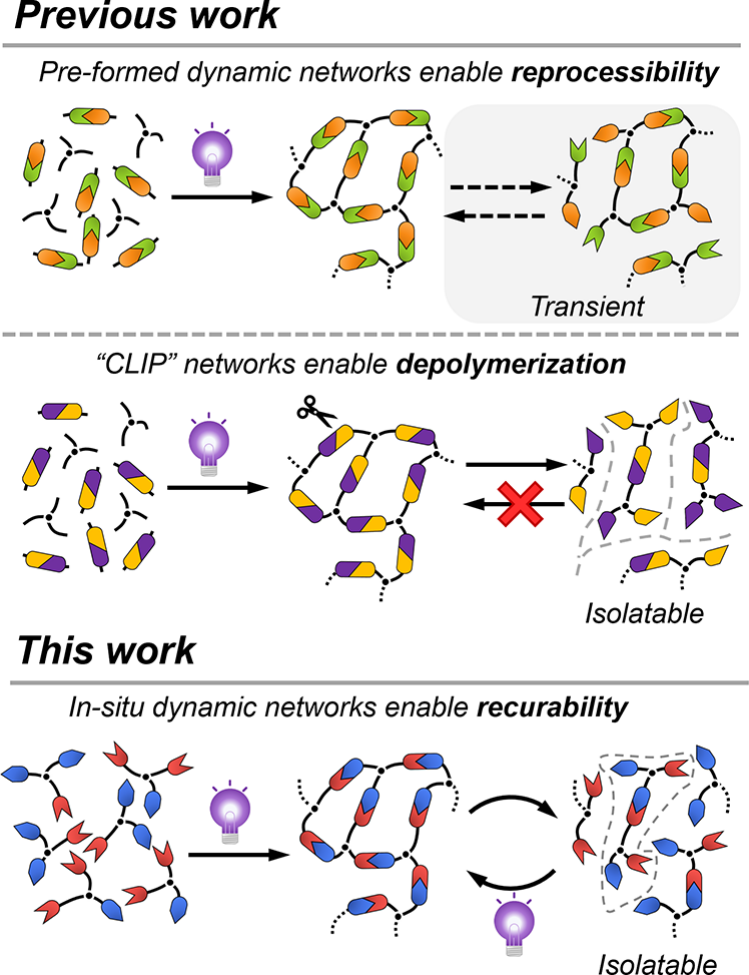
Previous work using the *pre-formed* dynamic network vs in situ formed dynamic network.

Conventional photopolymers undergo rapid, typically irreversible, covalent crosslinking upon exposure to a light stimulus, either by a photochemical coupling or radical initiation from a photoinitiator. The combination of on-demand, light-triggered fabrication and the robust properties of crosslinked structures have made photopolymers key to coatings, inks, and rapid prototyping technologies.^3−5^ Recent efforts to create more recyclable photopolymers have focused on the incorporation of dynamic bonds into structures, commonly referred to as covalent adaptable networks (CANs).^6^ Photopolymer CANs have been created in which, following photocuring, the transience of *pre-formed* dynamic bonds allow for networks to temporarily be thermally reformed into a new shape after application of a heating–cooling cycle. This approach is exemplified by the recent report from Bowman and co-workers, where the use of thiol-ene addition yielded a photocured material that contained *pre-formed* dynamic thiol-anhydride bonds.^7^ Another strategy incorporates the “CLIP” chemistry handle (covalent bonds that can undergo cleavage to form defined functionalities) into photopolymerizable neworks.^8^ Once the photopolymer is cured, the network can be depolymerized by degradation of the “CLIP” bonds. Labile ester, imine, or thioester bonds have been used most frequently in this context where network deconstruction is achieved by simple hydrolysis or transesterification reactions.^8−10^ Notably, in these examples, the depolymerization products from the photoset networks generate functionalities unsuitable for subsequent photopolymerization and require synthetic modification or additional reactive diluents to be reused as a photopolymer (Figure 1).^9,10^

While CAN and “CLIP” technologies mark a considerable milestone in developing more recyclable photopolymers, accessing photocurable networks that can be recycled back into a resin to re-fabricate a photoset network in a closed-loop process remains a challenge. To address this challenge, materials are required in which the reversible, or “CLIP”, bond is produced in situ rather than *pre-formed* in the network precursors. Therefore, the fabricated network can be depolymerized to regenerate the original functionalities and enable repeat photopolymerization (Figure 1). The closest examples to this aim utilize reversible cycloadditions, such as anthracene [4 + 4]^11^ and coumarin [2 + 2] dimerization^12^ as well as triazolinedione-naphthalene [2 + 4] reactions.^13^ However, these systems typically require high frequency (<300 nm) and/or continuous light irradiation, which can be costly, energy intensive and can generate undesirable side products.

**Figure 2:**
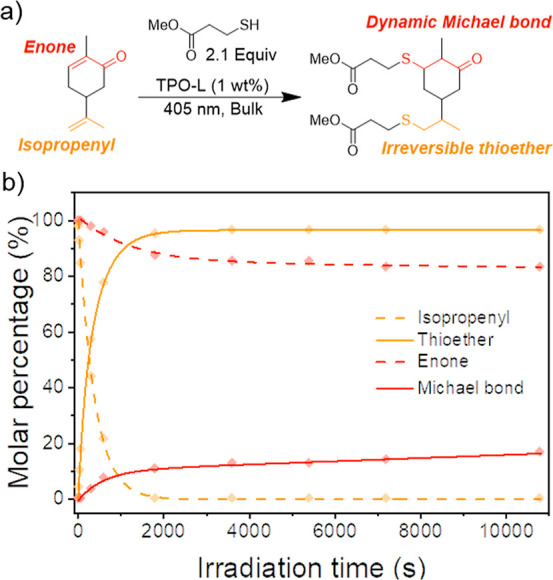
(a) Scheme of the radical-mediated addition of MMP to l-carvone using the radical initiator TPO-L (1 wt%) under 405 nm UV irradiation. (b) Plot of the molar percentage of each functionality over irradiation time monitored by ^1^H NMR spectroscopy. Lines represent a best fit.

To address this challenge, we envisioned a photopolymer platform in which orthogonal reactivity could be leveraged to furnish a network that could be reversibly cleaved on-demand to reform the original photoactive functionalities. The radical-mediated thiol-ene reaction was targeted as an optimal chemistry to achieve this target, as a consequence of its extensive use in photopolymer resins and the various well-established dynamic sulfur-based bonds.^14,15^l-carvone is a bio-sourced, inexpensive ($0.08/g), and commercially available compound that contains both a bench-stable tri-substituted enone moiety and another di-substituted alkene (or the isopropenyl group). These double bonds can react orthogonally under the radical thiol-ene conditions intended in the photopolymer platform (Figure 2a). While radical thiyl addition to isopropenyl groups creates irreversible thioethers, electron-deficient β-thioethers (or Michael bonds) have been established to undergo retro-Michael addition and reform the alkene-containing Michael acceptor and the thiol functionality.^16^ The majority of reports feature the enone functionality as a suitable Michael acceptor which has been shown to reverse at elevated temperatures and/or under basic conditions. However, synthetic enone species typically suffer with poor stability and require low atom economy, multistep syntheses to fabricate.^17^ Use of l-carvone as a bio-sourced building block for photopolymer resins not only offers a cost-effective solution,^18^ but also shifts reliance from traditionally used oil derivatives to a more sustainable source.

## Results and Discussion

Initially, we assessed the suitability of l-carvone to form a recyclable photopolymer resin platform with a series of model reactions. l-Carvone and methyl 3-mercaptopropionate (MMP) were mixed in bulk with a photoinitiator (TPO-L, 1 wt%) and exposed to UV light to simulate photopolymerization conditions (Figure 2a). A near-visible (λ = 405 nm) UV light was used to slow the reaction kinetics that could be readily measured due to the diminished absorption of TPO-L at that wavelength (Figure S1). The reaction was periodically sampled to quantify the consumption of both alkenes via ^1^H-NMR spectroscopy. Unsurprisingly, the less sterically hindered isopropenyl group was consumed faster and achieved a higher conversion (99% after 30 min) than the enone, which was significantly slower and achieved a much lower overall conversion (17% after 180 min, Figure S2 and Table S1). While the steric barrier of the tri-substituted enone hindered addition, the stabilizing effect of the ketone and the α-methyl group also contributed to its low reaction rate and overall conversion.^19,20^ Nevertheless, the observed reactivity of the enone highlighted its suitability within a photopolymer system.

The reversibility of the system via the thiol-Michael adduct depended on the formation of the β-ketone thioether; significant formation of the Markovnikov product (α-ketone thioether) could potentially limit the repeat recyclability of the network. Fortunately, the radically mediated thiol-ene reaction typically favors the anti-Markovnikov adduct, which would yield the desired β-ketone thioether for the l-carvone structure. Further analysis of the model reaction products was undertaken to probe this selectivity. Hetereonuclear single-quantum correlation spectroscopy was used to identify diagnostic peaks in the ^1^H-NMR spectra of the model compounds that are associated with the desired β-ketone thioether Michael adduct (Figure S3). Production of the desired β-ketone thioether corresponded with the concomitant consumption of the enone moiety, which indicates that the desired Michael bond is favored (Figure 2b).

**Figure 3:**
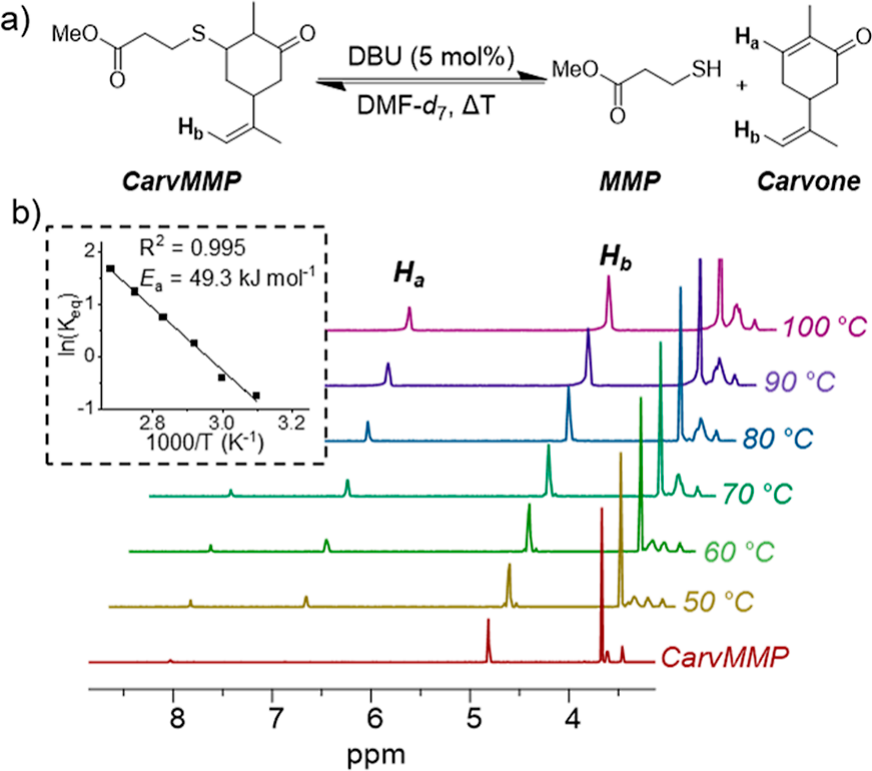
(a) Scheme for the dissociation of CarvMMP in the presence of DBU. (b) Variable temperature ^1^H-NMR of CarvMMP in DMF-*d*_7_ with DBU (5 mol%) illustrating the regeneration of the enone functionality at increasing temperature. Inset Van’t Hoff plot of the dissociation of CarvMMP.

Typically, reversible bonds, such as the Michael bonds, have been avoided in networks due to concerns that the dynamic network could behave similarly to a thermoplastic, that is, flowing at high temperatures and/or solubilizing in organic solvents.^21,22^ Although more recent work has suggested that reversible networks can be equally robust,^23^ we confirmed the reversibility of the Michael bond by extending our model study. To enable this study, a mono-functional thiol-Michael adduct (CarvMMP) was synthesized by the base-mediated conjugate addition of MMP to l-carvone. CarvMMP displayed no dissociation in DMF-*d*_7_; however, upon addition of DBU (5 mol%), dissociation at room temperature occurred rapidly, even before a single time point could be taken (Figure 3a). Further time points taken suggested that equilibrium was reached before the first time point (Figure S4) with variable temperature ^1^H-NMR spectroscopy studies (Figure 3b) enabling the determination of equilibrium constants at each temperature. Using these data, a Van’t Hoff plot (Figure 3b and Table S2) was constructed and revealed an activation energy of 49.3 kJ mol^–1^ for CarvMMP, which was comparable to previously reported dynamic Michael bonds.^16,24,25^ CarvMMP’s observed stability in the absence of DBU suggested that a carvone-based network would likely be robust at ambient temperature but could depolymerize readily upon addition of DBU at elevated temperature.

**Scheme 1:**
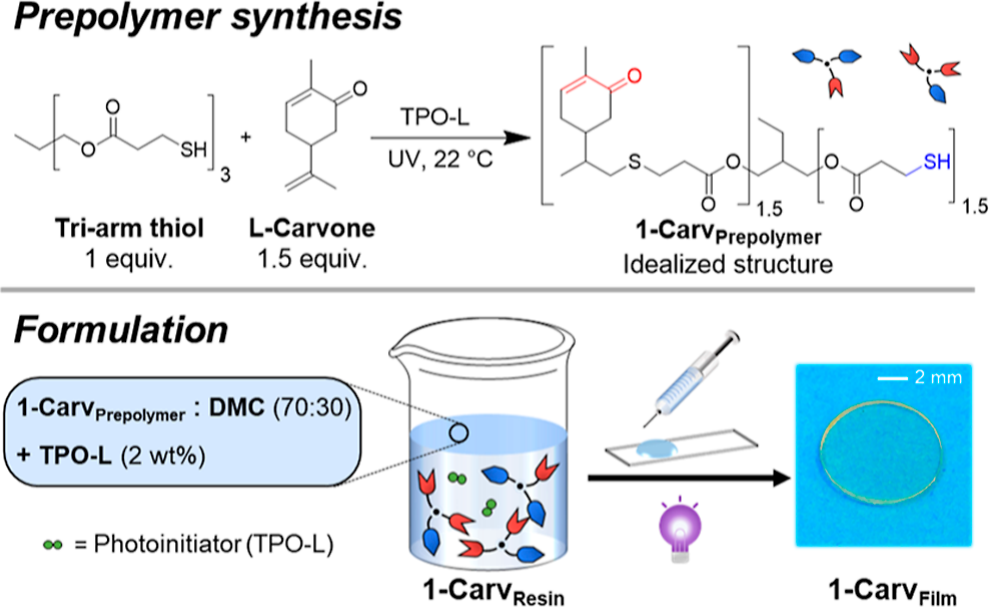
Schematic depiction of the thiol–ene reaction of l-carvone and trimethylolpropane tris(3-mercaptopropionate) to obtain the Carvone Prepolymer using TPO-L (1 wt%) Resin formulation and schematic diagram of the photopolymerization of 1-Carv_Resin_ into 1-Carv_Film_.

Extending these concepts to a photopolymer system was undertaken by creating a resin with a 3 + 2 network architecture in which the difunctional l-carvone and a three-arm thiol (trimethylolpropane tris(3-mercaptopropionate)) were combined at a molar ratio of 3:2, respectively, to ensure equimolar equivalents of alkene and thiol functionalities. A 3:2 mixture of l-carvone (1.5 equiv) and the three-arm thiol (1 equiv) was photocured, but the time to gelation was relatively slow (Figure S5). To improve the photocuring rate, we partially reacted the same mixture to form a prepolymer, which is known to reduce the time to gelation (Scheme 1).^26^ The considerable reactivity difference between l-carvone’s alkenes led to a total consumption of the isopropenyl functionality with only a partial consumption of the enone (6%) after 150 min of UV irradiation. As a result, the reaction could be quenched before crosslinking occurred, yielding a viscous liquid prepolymer, 1-Carv_Prepolymer_.

The photopolymer resin was formulated by diluting 1-Carv_Prepolymer_ with a non-reactive solvent to reduce the viscosity and improve handling (Figure S6). 1-Carv_Prepolymer_ was dissolved in dimethyl carbonate at a 70:30 weight ratio, respectively, and then the photoinitiator (TPO-L, 2 wt% with respect to the prepolymer component) was added to produce the initial resin, 1-Carv_Resin_. Upon irradiation with UV light, the resin transitioned from a viscous liquid to a freestanding solid structure, characteristic of a crosslinked polymer. Finally, the structure was post-cured in a vacuum oven at 90 °C to remove DMC and to maximize the crosslinking, which yielded a flexible network, 1-Carv_Film_ (Scheme 1).

**Figure 4:**
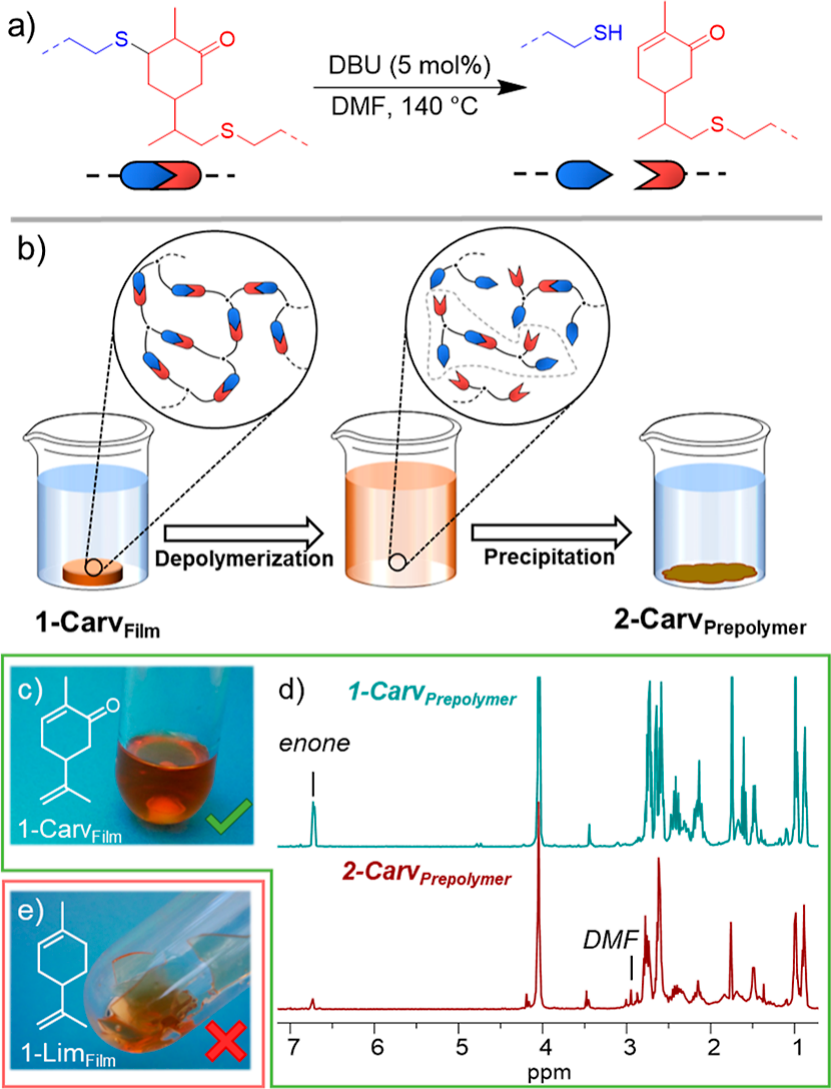
(a) Scheme illustrating the cleavage Michael bond in the presence of DBU (5 mol%) at 140 °C. (b) Diagram of the depolymerization of 1-Carv_Film_ to yield 2-Carv_Prepolymer_. (c) Image of 1-Carv_Film_ after depolymerization. (d) ^1^H-NMR spectra of 1-Carv_Prepolymer_ (top) and 2-Carv_Prepolymer_ (bottom). (e) Image of 1-Lim_Film_ after the depolymerization attempt.

We postulated that 1-Carv_Film_ could be depolymerized into soluble oligomers through retro-Michael addition at elevated temperature in the presence of a base to regenerate the enone and thiol functionality that are crucial for repeat UV curability of the structure. 1-Carv_Film_ was shredded and added into a degassed solution (to inhibit unwanted oxidation) containing DBU (5 mol%) with DMF (Figures 4a,b); the need of a high boiling point solvent was evident from the small molecule study. Dissolution of 1-Carv_Film_ readily occurred once a temperature of 140 °C was maintained for 4 h; however, the reaction was left for *ca.* 16 h to ensure that equilibrium had been reached (Figure 4c). Even though the vast majority of 1-Carv_Film_ had visibly dissolved, a small quantity of particulates could still be observed in the solution. These were assumed to be areas of high crosslink density that failed to depolymerize and only represented a small fraction of the initial material mass (*ca.* 0.5 wt%). Work-up of the depolymerization reaction required the removal of DBU and DMF, which can favor the forward Michael addition; hence, it was imperative to remove them before re-crosslinking could occur.^27^ Precipitation into methanol was found to be effective at removing undesired DBU and recover the majority (55%) of the initial network mass (Figure 4d). The non-quantitative mass recovery would ultimately limit the repeated recyclability of the photopolymer. However, using alternative precipitation solvents, such as water, can offer a greater mass recovery (71% recovery, Figure S7).

To evidence the necessity of the Michael bond in the depolymerization step, an analogous network that lacks the enone functionality was prepared. A network composed of limonene (1-Lim_Film_), an equivalent terpenoid that lacks the ketone functionality, and a tri-arm thiol were fabricated in an analogous manner to the carvone-based networks and subsequently subjected to the same depolymerization conditions. In contrast to 1-Carv_Film_, 1-Lim_Film_ remained visually intact under identical depolymerization conditions and displayed only a 1% weight loss (Figure 4e and the Supporting Information), which highlights the important role of the enone moiety in the carvone resin platform.

**Figure 5:**
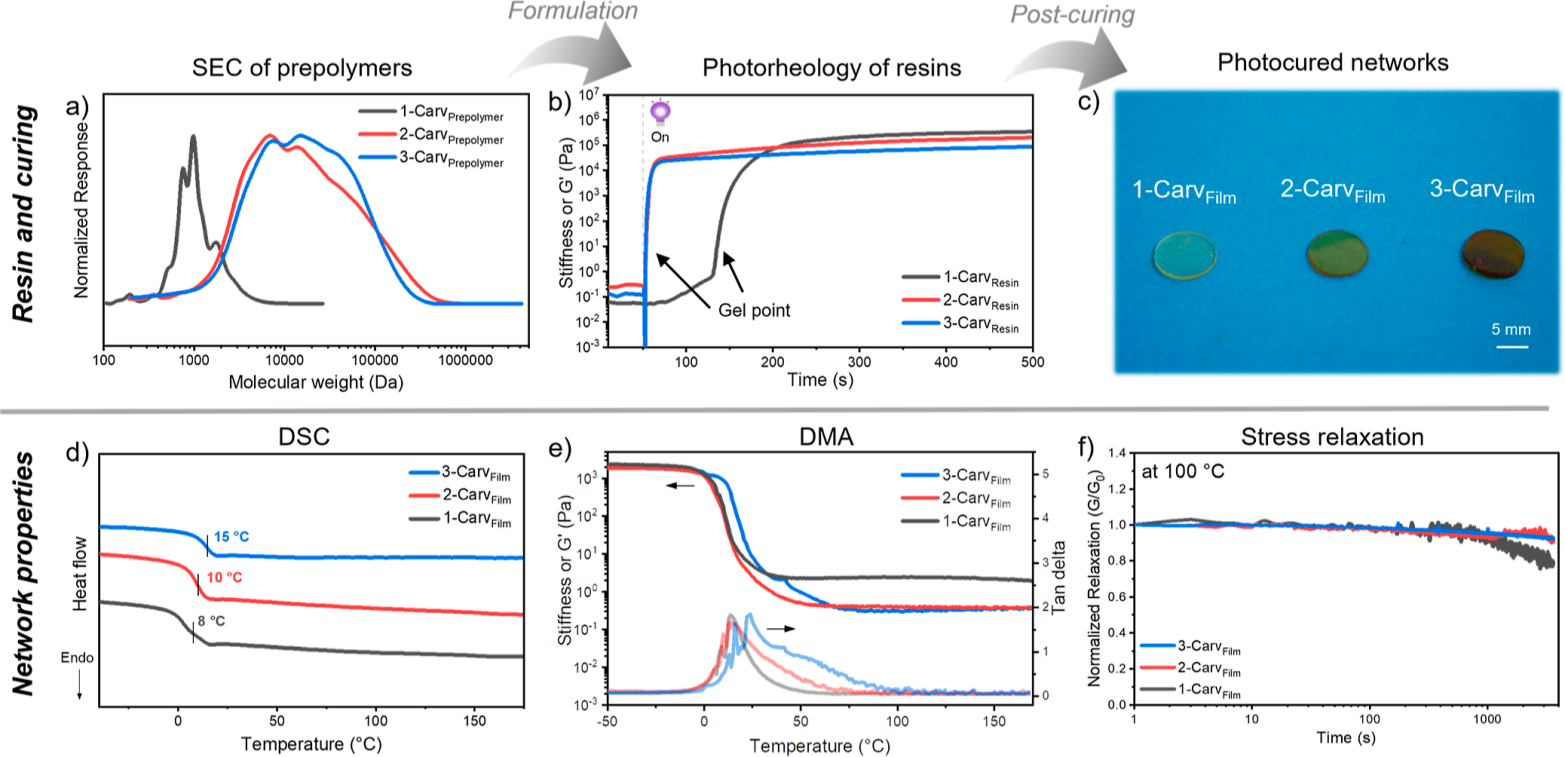
(a) SEC chromatograms of 1-Carv_Prepolymer_, 2-Carv_Prepolymer_, and 3-Carv_Prepolymer_ (CHCl_3_, v/v 2% NEt_3_) against polystyrene standards. (b) Photorheology of each resin system taken over 500 s under oscillatory shear at ambient temperature. (c) Image of 1-Carv_Film_, 2-Carv_Film_, and 3-Carv_Film_. (d) DSC thermograms of the second heating cycle for the initial and both re-cures. (e) DMA thermograms of storage modulus and tan delta vs temperature for the initial and both re-cures in the tensile configuration. (f) Normalized stress relaxation of 1-Carv_Film_, 2-Carv_Film_, and 3-Carv_Film_ at 100 °C and 2% strain taken over 3000 s.

Finally, the soluble oligomers (2-Carv_Prepolymer_) that were recovered from the depolymerization of 1-Carv_Film_ were analyzed and compared with the initial prepolymer, 1-Carv_Prepolymer_. ^1^H NMR spectroscopy confirmed that the enone had been recovered (31%), and the depolymerized product resembled the initial prepolymer (Figure 4c,d). Size exclusion chromatography (SEC) was also used to estimate the molecular weight of 2-Carv_Prepolymer_ and 1-Carv_Prepolymer_ (Figure 5a). As expected, 2-Carv_Prepolymer_ had a significantly higher molecular weight and dispersity (*M*_n_ = 5.8 kDa, D̵ = 6.3) than 1-Carv_Prepolymer_ (*M*_n_ = 0.9 kDa, D̵ = 1.4), as a consequence of the non-quantitative cleavage of the thiol-Michael bond.

After demonstrating that 1-Carv_Resin_ could be cured and depolymerized into 2-Carv_Prepolymer_ to regenerate the enone functionality, the recycled resin was re-formulated. As a consequence of the molecular weight variation, 2-Carv_Prepolymer_ required a higher dilution with DMC (55:45 weight ratio, respectively) than the initial 1-Carv_Prepolymer_ (70:30 weight ratio of prepolymer to DMC). The curing profiles of the recycled 2-Carv_Resin_ were assessed against the initial 1-Carv_Resin_ using photorheology. A low storage modulus was observed in both resins in their liquid phase, with a sudden increase in their stiffnesses upon exposure to UV light (Figure 5b). The storage modulus eventually reaches a plateau, characteristic of a sol–gel transition. 2-Carv_Resin_ also achieved gelation faster (4 s) than the initial 1-Carv_Resin_ (85 s), most likely as a result of the higher molecular weight species requiring fewer crosslinks to form an infinite network. This could be considered advantageous for more rapid photopolymerization of the recycled product. 2-Carv_Resin_ achieved a plateau at a marginally lower storage modulus than 1-Carv_Resin_; this was attributed to a comparatively lower crosslinking density. This was further supported by a solvent swelling experiment, where 2-Carv_Film_ had a higher degree of swelling in THF (315 ± 39% versus the initial cure 239 ± 3%) due to the increased distance between covalent crosslinks equating to a lower effective crosslinking density within 2-Carv_Film_. We hypothesized that the lower observed crosslinking density could arise from two different factors: unwanted thiol oxidation during depolymerization or the formation of loop defects which could reduce the network elasticity.^28^ Quantifying these factors can be particularly challenging, and a combination of different events are likely responsible for the observed lower crosslinking density in 2-Carv_Film_.^29^ Despite the small loss in crosslinking density between the initial and the recycled material, the reversibility of this carvone-based photopolymer without the use of reactive diluents or synthetic modification is a significant milestone.

Larger films of 1-Carv_Film_ and 2-Carv_Film_ were fabricated and post-cured to assess whether the bulk and thermal properties were comparable between recycles and displayed properties desirable from network materials. There was measurable discoloration of each prepolymer after depolymerization, which translated to color differences in photocured materials (Figures 5c and S8). This could be caused by the oxidation of sulfur species and/or degradation of the organobase catalyst during the depolymerization step. Despite the differences in physical appearance, both materials were found to possess a high thermal stability (*T*_d,5%_ > 295 °C) with an almost identical mass loss profile (Table S4 and Figure S20). The glass-transition temperatures (*T*_g_) of the post-cured networks were estimated using differential scanning calorimetry (DSC). Both 1-Carv_Film_ and 2-Carv_Film_ displayed a comparable *T*_g_, highlighting a good retention in the thermal characteristics for the recycled and the initial photopolymerized network (Figure 5d). To explore the mechanical properties of 1-Carv_Film_ and 2-Carv_Film_, dynamic mechanical analysis (DMA) was performed over a temperature ramp (Figure 5e). The peak observed in the tan delta is a characteristic transition relating to *T*_g_ and occurs at a similar temperature in both 1-Carv_Film_ and 2-Carv_Film_, which further supports the observation from the DSC thermograms. There is a notable broadening of the tan delta peak in 2-Carv_Film_, which is commonly attributed to heterogeneity in the network, but it is only marginally different from the initial cure.^30^ There was an observed decrease in the storage modulus for 2-Carv_Film_ in comparison to 1-Carv_Film_ within the DMA thermograms. A similar decrease in the ultimate tensile strength is also observed, but this is likely the result of the lower crosslink density in the recycled film (Figure S9 and Table S3).

However, the most distinctive property of a network polymer is dimensional stability and a high resistance to stress at elevated temperatures. This is evident in 1-Carv_Film_ and 2-Carv_Film_ from the presence of plateau in storage modulus (i.e., rubbery plateau) that persists after the *T*_g_ to high temperatures in the DMA thermograms (Figure 5e). Linear polymers and some CANs display a drop in the storage modulus after the *T*_g_ as the materials transition to liquid flow.^31^ Both 1-Carv_Film_ and 2-Carv_Film_ display a storage modulus plateau from 50 to 170 °C, exemplifying the excellent dimensional stability of both materials over that temperature range. This is further evidenced by the high resistance to stress relaxation of 1-Carv_Film_ and 2-Carv_Film_ at 100 °C (Figure 5f). Unusually, 1-Carv_Film_ displayed the least resistance to stress despite possessing the highest crosslinking density. This is likely the result of a higher relative concentration of dynamic Michael bonds, which facilitates stress relaxation in 1-Carv_Film_ as compared to 2-Carv_Film_.^32^

Demonstrating the repeat photopolymerization of the l-carvone resin system is a significant achievement within itself; however, true reversibility must be repeatable on the same material while maintaining the same mechanical robustness. Hence, 2-Carv_Film_ was subjected to the same depolymerization conditions and work-up as the initial carvone network. Almost total dissolution of the network was observed, with the same trivial quantities of the insoluble particulates (*ca.* 0.1 wt%) observed in the reaction mixture. Precipitation of the reaction yielded 3-Carv_Prepolymer_ with a similar enone and mass recovery (mass recovery = 57% and enone recovery = 19%, Table S5) and a similar molecular weight and distribution (*M*_n_ = 6.0 kDa, D̵ = 5.2) as 2-Carv_Prepolymer_. Dilution of 3-Carv_Prepolymer_ in DMC (55:45 weight ratio, respectively) with a photoinitiator (TPO-L, 2 wt% with respect to the prepolymer component) yielded 3-Carv_Resin_. Following this, the curing profile of 3-Carv_Resin_ was established using photorheology and found to have a comparable gelation time (4 s) to 2-Carv_Resin_ (Figure 5b). Unusually, there is a significant increase in *T*_g_ for 3-Carv_Film_ versus 1-Carv_Film_ and 2-Carv_Film_, despite its lower crosslinking density (Table S4). The simultaneous broadening of the tan delta peak can also be observed with each subsequent photopolymerization, which could be attributed to increased network heterogeneity or a loss of the low molecular weight species during precipitation (Figure 5e).^33^ Despite these observed differences, the tensile properties were found to be comparable between 2-Carv_film_ and 3-Carv_film_ (Figure S9 and Table S3), which highlights an excellent retention of bulk properties in the second recycle of this photopolymer system.

## Conclusions

We have designed a re-curable photopolymer platform based on radical-mediated thiol-ene reactions of a commodity terpenoid chemical, l-carvone. The depolymerization and re-curing of the carvone-based network was verified for three cycles, and the robust mechanical properties, illustrative of crosslinked polymers, were retained for each cycle. However, there are several caveats (non-quantitative mass recovery and structural heterogeneity) that diminish the “drop-in” application of the presented materials in this study. Our current efforts are focused on mitigating material loss during recycling to improve the overall efficiency of the closed-loop system and examining the morphology of recycled samples to better explain differences in bulk properties. Nevertheless, the simplicity and potential modularity of the carvone platform offers translation to the ever-growing number of applications reliant on photopolymers while simultaneously inspiring a transition to sustainable feedstock chemicals.

## Supporting Information Available

Full synthetic and technical procedures including thermal and mechanical characterization (PDF)

## Abbreviations

CAN: covalent adaptable network
MMP: methyl 3-mercaptopropionate
DMC: dimethyl carbonate
*M*_n_: number-average molar mass
NMR: nuclear magnetic resonance
GPC: gel permeation chromatography
DSC: differential scanning calorimetry
DMA: dynamic mechanical analysis
DBU: 1,8-diazabicyclo(5.4.0)undec-7-ene
DMF: *N*,*N*-dimethylformamide
*T*_g_: glass-transition temperature
TGA: thermal gravimetric analysis
UV: ultraviolet
