# Supplementary material for: Intrinsically Re-curable Photopolymers Containing Dynamic Thiol-Michael Bonds
Source: J Am Chem Soc. 2022 Jun 24;144(26):11729–35. doi: 10.1021/jacs.2c03525 (PMC9264357; doi:10.1021/jacs.2c03525)
Supplement: Supplementary file 1 — ja2c03525_si_001.pdf [file ja2c03525_si_001.pdf]

Intrinsically re-curable photopolymers containing dynamic thiol-Michael bonds

Connor J. Stubbs, Anissa L. Khalfa, Viviane Chiaradia, Joshua C. Worch, Andrew P. Dove*

School of Chemistry, University of Birmingham, Birmingham, B15 2TT, UK

Corresponding Authors: adove@bham.ac.uk

General Materials and Methods 2

Experimental Procedures 5

UV-Vis spectrum of TPO-L 10

Thiol-ene kinetic experiments 11

Identifying ^{1}H NMR peaks associated to the Michael product 12

Dynamic exchange experiment at ambient temperature 13

Van't Hoff plot 14

Improved weight recovery through precipitation into distilled water 15

Comparison of ^{1}H NMR spectra of the depolymerized networks 20

NMR Spectra 21

Differential Scanning Calorimetry thermograms 25

Thermogravimetric Analysis Data 26

Fourier Transform Infrared spectra of prepolymers and carvone networks 27

Photorheology 28

Dynamic Mechanical Analysis Thermograms 29

Stress relaxation experiments 30

Thermomechanical properties summary 31

Prepolymer summary 32

General Materials and Methods

All compounds, unless otherwise indicated, were purchased from commercial sources, and used as received. Methyl 3-mercaptopropionate was distilled and stored under a nitrogen atmosphere in an ampoule.

NMR Spectroscopic Analysis. All NMR spectroscopy experiments were performed at 300 K on a Bruker DPX-400 NMR instrument equipped with a BBFO smart probe operating at 400 MHz for ^{1}H (100.57 MHz for ^{13}C). ^{1}H NMR spectra are referenced to solvent residual proton (δ = 7.26 for $CDCl_3$, δ = 2.91 for DMF-d_7) and ^{13}C NMR spectra are referenced to the solvent signal (δ = 77.16 for $CDCl_3$, δ = 162.7 for DMF-d_7). The resonance multiplicities are described as s (singlet), d (doublet), t (triplet), q (quartet) or m (multiplet).

Mass Spectrometry. High Resolution Electrospray Ionization Mass Spectrometry was performed in the School of Chemistry at University of Birmingham on a Waters Xevo G2-XS QTof Quadrupole Time-of-Flight mass spectrometer.

UV-Vis spectroscopy. UV-Vis spectroscopy was performed on an Evolution 350 UV-Vis spectrophotometer equipped with Xenon Flash Lamp light source and Dual Matched Silicon Photodiodes detector. Quartz cells (170 - 2000 nm) from Hellma with two polished sides were used for examining the absorption spectra between 335 to 800 nm. Thermo INSIGHT-2 v.10.0.30319.1 software was used for data acquisition and analysis.

Size Exclusion Chromatography (SEC). SEC measurements were performed in $CHCl_3$ on an Agilent 1260 Infinity II Multi-Detector SEC System fitted with RI, ultraviolet (UV, λ = 309 nm), and viscometer detectors. The polymers were eluted through an Agilent guard column (PLGel 5 μM, 50 × 7.5 mm) and two Agilent mixed-C columns (PLGel 5 μM, 300 × 7.5 mm) using $CHCl_3$ (buffered with 0.5% NEt_3) as the mobile phase (flow rate = 1 mL·min^{-1}, 40 °C). Number average molecular weights (M_n), weight average molecular weights (M_w) and dispersities ($Đ_M = M_w/M_n$) were determined using Agilent GPC software (vA.02.01) against a 15-point calibration curve (M_p = 162 – 3,187,000 g·mol^{-1}) based on poly(styrene) standards (Easivial PS-M/H, Agilent).

Differential Scanning Calorimetry (DSC). The thermal characteristics of the polymers were determined using differential scanning calorimetry (STARe system DSC3, Mettler Toledo) from −50 to 180 °C at a heating rate of 10 °C·min^{-1} for two heating/cooling cycles unless otherwise specified. The glass transition temperature (T_g) was determined from the inflection point in the second heating cycle of DSC.

Thermogravimetric Analysis (TGA). TGA thermograms were obtained using a Q550 Thermogravimetric Analyzer (TA instruments). Thermograms were recorded under an N_2 atmosphere at a heating rate of 10 °C·min^{-1}, from 10 to 600 °C, with an average sample weight of *ca*. 5 mg. Aluminium pans were used for all samples. Decomposition temperatures were reported as the 5% weight loss temperature ($T_{d,5\%}$).

Fourier-transform infrared (FTIR) spectroscopy. FTIR spectra were collected out using an Agilent Technologies Cary 630 FTIR spectrometer. 16 Scans from 600 to 4000 cm^{-1} were taken at a resolution of 2 cm^{-1}, and the spectra were corrected for background absorbance.

Rheology. Resin viscosity values were obtained from shear rate sweeps from 0.1 to 100 s^{-1} on an Anton Paar MCR 302 using with a PP25 geometry. Resins were loaded onto the plate at ambient temperature with a gap of 0.5 mm.

Stress-Relaxation was performed on an Anton Paar MCR-302 using Anton Paar PP8 parallel-plate, with a diameter of 8 mm. Temperature was controlled with a P-PTD 200/AIR Peltier and a P-PTD 200 hood. Strain sweeps were performed to ensure stress relaxation experiments were performed in the linear region. Stress relaxation tests were performed at 2% strain at 100 °C and 140 °C. ($n = 1$)

Photorheology. The crosslinking kinetic of the resins was examined as a function of gelation time by photorheology using an Anton Paar MCR-302 rheometer fitted with a detachable photoillumination system (Exfo OmniCure S1500 UV light source, broadband Hg-lamp, glass plate). Resin samples were sheared between two parallel plates at 0.2 Hz with an amplitude of 25% for 50 s without irradiation. After this time, the light source was switched on and measurements were taken every 0.2 s over the course of 450 s. The intersection point of the storage moduli and loss moduli plots was used to determine the time of gelation of the resin.

Dynamic Mechanical Analysis (DMA). Dynamic mechanical thermal analysis (DMTA) data were obtained using a Mettler Toledo DMA 1 star system and analyzed using the software package STARe V13.00a Thermal sweeps were conducted using films (L x W x thickness = 15 mm x 6 mm x 0.5 mm) cooled to −50 °C and held isothermally for *ca*. 5 minutes. Storage and loss moduli, as well as the loss factor (ratio of E'' and E', tan δ) were probed as the temperature was swept from −50 to 180 °C, 5 °C·min^{-1}. Thermomechanical behavior was determined from three samples in this way. ($n = 3$, unless otherwise specified)

Tensile testing. Uniaxial tensile testing was performed using a Testometric M350-5CT universal mechanical testing instrument fitted with a load cell of 10 kN. Film samples were visually inspected for deformation and bubbles then dumbbell shaped samples were cut using custom ASTM Die D-638 Type 5. Each specimen was clamped into the tensile holders and subjected to an elongation rate of 10 mm·min^{-1} until failure. All tensile tests were repeated 3 times, and an average of the data was taken to find the ultimate tensile stress and strain. Data was analyzed using winTest™ Analysis software (v.5.0.34) and OriginPro® software.

Swell testing and gel fraction. Networks were taken and cut into disks (6.7 × 0.5 mm) and weighed before being submerged in THF and allowed to swell until equilibrium swelling was reached after 24 hours. The initial mass (W_i) and swelling mass (W_s) was used to calculate the swelling ratio (%) as shown in equation S1. The swollen networks were resubmerged in new THF and finally dried to get the dry mass (W_d). This was used to calculate the gel fraction (%) as shown in equation S2.

Equation S1 $$\boldsymbol{Swelling\ ratio}\ (\%) = \frac{\boldsymbol{W_s}}{\boldsymbol{W_i}} \times \mathbf{100}$$

Equation S2 $$\boldsymbol{Gel\ fraction}\ (\%) = \frac{\boldsymbol{W_d}}{\boldsymbol{W_i}} \times \mathbf{100}$$

Experimental Procedures

Synthesis of CarvMMP

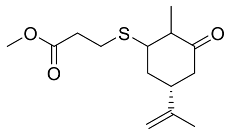

L-carvone (2.00 g, 1 equiv, 13.3 mmol) and methyl 3-mercaptopropionate (1.76 g, 1.1 equiv, 14.7 mmol) were mixed in bulk at 0 °C until homogenous. DBU (19.5 µL, 1 mol%) was then added in one-portion and the reaction was left to stir at 0 °C for 30 min before warming to ambient temperature. The mixture was purified using silica column chromatography with EtOAc/Hexanes and concentrated yielding a colorless oil (1.9 g, 50%). ^{1}H-NMR (400 MHz, Chloroform-*d*) δ 4.85 – 4.65 (m, 2H), 3.67 (s, 3H), 3.43 (dt, J = 4.7, 3.4 Hz, 1H), 2.94 – 2.82 (m, 1H), 2.82 – 2.70 (m, 3H), 2.64 – 2.50 (m, 2H), 2.46 (ddd, J = 13.7, 4.4, 2.1 Hz, 1H), 2.26 – 2.11 (m, 2H), 1.99 (ddd, J = 13.8, 11.8, 3.2 Hz, 1H), 1.74 (s, 3H), 1.12 (d, J = 6.7 Hz, 3H). ^{13}C NMR (101 MHz, Chloroform-*d*) δ 209.67, 172.25, 147.04, 110.38, 51.93, 50.30, 48.75, 46.10, 40.75, 35.96, 34.68, 27.10, 20.89, 12.71. ^{1}H-NMR (400 MHz, DMF-d_7) δ 4.80 (s broad, 2H), 3.65 (s, 3H), 3.60 (dt, *J* = 4.9, 3.3 Hz, 1H), 3.09 – 2.98 (m, 1H), 2.89 – 2.76 (m, 3H), 2.70 – 2.57 (m, 2H), 2.45 (t, J = 13.2 Hz, 1H), 2.24 (dd, J = 13.4, 4.0 Hz, 1H), 2.20 – 2.06 (m, 2H), 1.77 (s, 3H), 1.04 (d, J = 6.7 Hz, 3H). ^{13}C-NMR (101 MHz, DMF-d_7) δ 209.56, 172.59, 148.39, 110.27, 51.77, 50.82, 48.41, 46.40, 41.53, 36.31, 35.01, 27.19, 20.60, 12.76. MS (TOF) calculated for $C_{14}H_{22}O_3S$ + H 271.1368; found 271.1374.

Radical addition of MMP to carvone model reaction

L-carvone (0.8513 g, 1 equiv, 5.68 mmol) and methyl 3-mercaptopropionate (1.4303 g, 2.1 equiv, 11.9 mmol) were mixed in bulk until visibly homogenous in a glass vial. TPO-L (0.022 g, 1 wt%) was then added in one portion and vigorously stirred. The mixture was then irradiated with UV light in a Formlabs Form Cure 405 nm curer and sampled periodically (10 s, 30 s, 60 s, 5 min, 10 min, 30 min, 1 h, 1.5 h, 2 h, 3 h) for ^{1}H-NMR spectroscopy. Data was plotted against irradiation time and lines of best fit were generated in OriginPro 2019 (9.6.0.172) using the exponential decay fitting function.

Dynamic exchange model reaction ambient temperature

CarvMMP (135 mg, 0.5 mmol) was dissolved in DMF-d_7 (2 mL) in a glass vial, then DBU (3.7 µL, 5 mol%) was added and stirred rapidly. An aliquot was taken for an initial ^{1}H NMR spectrum. The remaining solution was left stirring at ambient conditions and sampled periodically (30 min, 1 h, 2 h) for ^{1}H NMR spectroscopy.

Dynamic exchange model reaction variable temperature NMR

CarvMMP (67.5 mg, 0.25 mmol) was dissolved in DMF-d_7 (0.5 mL) and added to an NMR tube. A reference ^{1}H NMR spectrum was taken at 298 K. DBU (1.9 µL, 5 mol%) was dissolved in DMF-d_7 (0.5 mL) and added to the NMR tube then briefly stirred. ^{1}H NMR spectrums were taken at various temperatures (50, 60, 70, 80, 90, 100 °C) after a 5-minute equilibration time at each temperature.

Synthesis of 1-Carv$_{Prepolymer}$

L-carvone (10 g, 1.5 equiv, 66.7 mmol) and trimethylolpropane tris(3-mercaptopropionate) (17.68 g, 1 equiv, 44.4 mmol) were mixed in bulk until visibly homogenous in a 100 mL round bottom flask (RBF) with a magnetic stirrer. The RBF was wrapped in foil, then TPO-L (0.277 g, 1 wt%) was added and stirred for *ca.* 5 min until homogenous. The RBF was partially exposed by ripping the foil and irradiated with UV light using a fiber optic cable fitted to an OmniCure S1500 UV light source and stirred for *ca.* 2 h at ambient temperature. Progress of the reaction was monitored by ^{1}H-NMR spectroscopy for the disappearance of the propenyl group. The resulting colorless oil was used as the 1-Carv$_{Prepolymer}$. ^{1}H-NMR (400 MHz, Chloroform-*d*) δ 6.75 (s, broad 1H), 4.08 (s, overlapping 6H), 2.91 – 2.56 (m, overlapping, 14H), 2.54 – 2.27 (m, overlapping, 5H), 2.27 – 1.99 (m, overlapping, 5H), 1.78 (s, J = 1.4 Hz, 4H), 1.76 – 1.55 (m, overlapping, 3H), 1.59 – 1.33 (m, 2H), 1.01 (d, J = 6.8 Hz, 4.3H), 0.91 (t, J = 6.5 Hz, 3H). ^{13}C NMR (101 MHz, $CDCl_3$) δ 199.96, 171.54, 171.32, 63.96, 42.58, 40.84, 40.63, 39.29, 39.19, 38.45, 37.28, 37.15, 37.07, 34.80, 30.52, 28.41, 27.75, 27.67, 23.04, 19.77, 16.01, 15.78, 7.47. GPC analysis ($CHCl_3$ + 0.5% v/v NEt_3): M_w = 1.2 kDa, M_n = 0.9 kDa, $Đ$ = 1.4. FT-IR: 1726 cm^{-1} (C=O ester) 1659 cm^{-1} (C=O enone).

Depolymerization of 1-Carv$_{Film}$ to produce 2-Carv$_{Prepolymer}$

Shredded 1-Carv$_{Film}$ (10.55 g, 1 equiv, 16.9 mmol), DMF (67 mL) and DBU (126 µL, 5 mol%) were added to an ampoule with a magnetic stirrer and stirred at ambient temperature. Nitrogen is bubbled through the mixture for ~ 30 min before the flask is sealed and heated at 140 °C for *ca.* 16 h. The reaction is removed from the heat and filtered through fiberglass wool to remove the insoluble particulates, the residue is dried and weighed to assess the quantity of particulates (49 mg, 0.5% of the original weight). The filtrate is precipitated into methanol (~800 mL), centrifuged and the supernatant is discarded. The remaining orange tacky solid is washed with additional methanol (~200 mL) and then the supernatant was discarded. Residual solvent is removed under vacuum for 2 h to yield a tacky orange solid 2-Carv$_{Prepolymer}$ (5.80 g, 55%). GPC analysis ($CHCl_3$ + 0.5% v/v NEt_3): M_w = 36.1 kDa, M_n = 5.8 kDa, $Đ$ = 6.3. FT-IR: 1727 cm^{-1} (C=O ester) 1697 cm^{-1} (C=O ketone) 1667 cm^{-1} (C=O enone)

Depolymerization of 2-Carv$_{Film}$ to produce 3-Carv$_{Prepolymer}$

Shredded 2-Carv$_{Film}$ (3.28 g, 1 equiv, 5.26 mmol), DMF (21 mL) and DBU (39 µL, 5 mol%) were added to an ampoule with a magnetic stirrer and stirred at ambient temperature. Nitrogen is bubbled through the mixture for ~ 30 min before the flask is sealed and heated at 140 °C for *ca* 16 h. The reaction is removed from the heat and filtered through fiberglass wool to remove the insoluble particulates, the residue is dried and weighed to assess the quantity of particulates (3.7 mg, 0.1% of the original weight). The filtrate is precipitated into methanol (~350 mL), centrifuged and the supernatant is discarded. The remaining orange tacky solid is washed with additional methanol (~100 mL) and then the supernatant was discarded. Residual solvent is removed under vacuum for 2 h to yield a tacky orange solid 3-Carv$_{Prepolymer}$ (1.87 g, 57%) GPC analysis ($CHCl_3$ + 0.5% v/v NEt_3): M_w = 31.1 kDa, M_n = 6.0 kDa, $Đ$ = 5.2. FT-IR: 1724 cm^{-1} (C=O ester) 1697 cm^{-1} (C=O ketone) 1666 cm^{-1} (C=O enone).

Depolymerization of 1-Carv$_{Film}$ and precipitation into distilled water

Shredded 1-Carv$_{Film}$ (0.6508 g, 1 equiv, 1.0 mmol), DMF (4.2 mL) and DBU (7.7 µL, 5 mol%) were added to an ampoule with a magnetic stirrer and stirred at ambient temperature. Nitrogen is bubbled through the mixture for ~ 30 min before the flask is sealed and heated at 140 °C for *ca.* 16 h. The reaction is removed

from the heat and filtered through fiberglass wool to remove the insoluble particulates. The filtrate is precipitated into water (~80 mL), centrifuged and the supernatant is discarded. The remaining mixture is freeze-dried to remove residual water to yield a tacky orange solid 2-CarvPrepolymer (0.4653 g, 71%). GPC analysis ($CHCl_3$ + 0.5% v/v NEt_3): M_w = 9.0 kDa, M_n = 1.9 kDa, $Đ$ = 4.7.

Attempted depolymerization of 1-Lim_{Film}

Shredded 1-Lim_{Film} (0.7377 g, 1 equiv, 1.2 mmol), DMF (4.9 mL) and DBU (9 µL, 5 mol%) were added to an ampoule with a magnetic stirrer and stirred at ambient temperature. Nitrogen is bubbled through the mixture for ~ 30 min before the flask is sealed and heated at 140 °C for *ca.* 16 h. The reaction is removed from the heat and filtered through fiberglass wool to remove the unreacted 1-Lim_{Film}, the residual material is dried and weighed to assess the mass loss 0.7298 g, 99% of the original weight (or 1% weight loss).

Formulation of 1-$Carv_{Resin}$

1-$Carv_{Prepolymer}$ (7 g, 70 wt ratio) was added to a vial and stirred in dimethylcarbonate (3 g, 30 wt ratio) until the liquid was homogenous. The vial was wrapped in foil and TPO-L (0.14 g, 2 wt%) was added, then the vial was agitated using an orbital shaker for 1 h until the liquid was homogenous.

Formulation of 2-$Carv_{Resin}$

2-$Carv_{Prepolymer}$ (4.94 g, 55 wt ratio) was added to a vial and stirred in dimethylcarbonate (4.04 g, 45 wt ratio) until the liquid was homogenous. The vial was wrapped in foil and TPO-L (0.0988 g, 2 wt%) was added, then the vial was agitated using an orbital shaker for 1 h until the liquid was homogenous.

Formulation of 3-$Carv_{Resin}$

3-$Carv_{Prepolymer}$ (1.87 g, 55 wt ratio) was added to a vial and stirred in dimethylcarbonate (1.53 g, 45 wt%) until the liquid was homogenous. The vial was wrapped in foil and TPO-L (0.0374 g, 2 wt%) was added, then the vial was agitated using an orbital shaker for 1 h until the liquid was homogenous.

Formulation of 1-Lim$_{Resin}$

Limonene (2 g, 1.5 equiv), trimethylolpropane tris(3-mercaptopropionate) (3.90 g, 1 equiv, 44.4 mmol) and dimethylcarbonate (1.47 g, 20 wt ratio) were stirred in a vial until homogenous. TPO-L (0.03 g, 2 wt%) was added to the mixture and rapidly stirred until dissolved. The resulting colorless liquid was cured in the same manner as the carvone resins, as described in the general curing procedure.

General resin curing procedure

Resins were deposited on a glass slide using a glass pipette to ensure a flat surface and even coverage. The glass slide is positioned above the UV light source (an OmniCure S1500 fitted with a fiber optic cable) and irradiated from beneath the glass slide at approx. 15 cm. *Note: positioning the light source below the glass slide ensured thick films did not exhibit surface wrinkling and defects over extended curing periods.* The resins were inspected periodically until the surface of the film was solid and non-tacky (typically ~1 hour for 1 mm thickness). The films were removed from the glass slide and post-cured in a vacuum oven at 90 °C under reduced pressure for *ca.* 16 h to remove dimethyl carbonate and ensure the network was sufficiently crosslinked.

UV-Vis spectrum of TPO-L

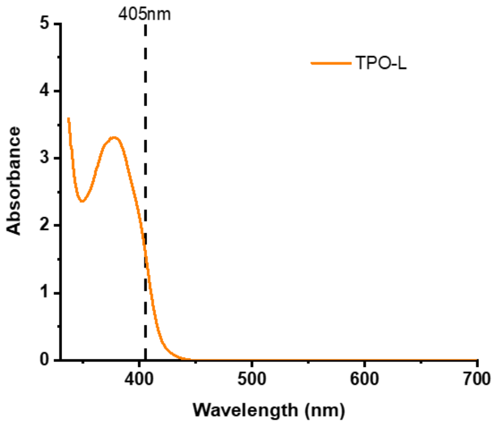

Figure S1. UV-Vis spectrum of TPO-L in NMP with vertical line illustrating absorption at 405 nm.

Thiol-ene kinetic experiments

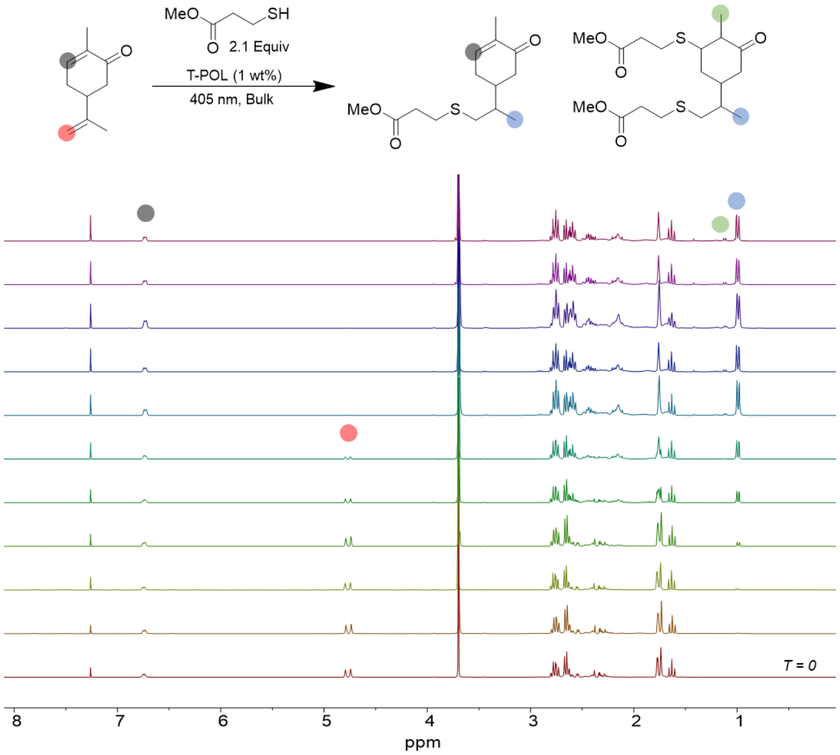

Figure S2. ^{1}H NMR spectra of the addition of MMP to L-carvone *via* radical mediated thiol-ene at increasing radiation times in $CDCl_3$. Consumption and formation of each functionality was referenced against the methyl ester singlet at 3.69 ppm and summarized in Table S1.

Table S1. Molar percentage of the monitored functionalities in the radical addition of MMP to L-carvone against UV irradiation time.

Irradiation time (s)	Enone (%)	Propenyl (%)	Thioether (%)	Michael bond (%)
0	100	100	0	0
10	100	98	4	0
30	100	93	10	0
60	100	85	18	0
300	98	44	57	3
600	96	21	78	7
1800	88	0	95	11
3600	85	0	97	13
5400	85	0	97	13
7200	83	0	97	14
10800	83	0	97	17

Identifying ^{1}H NMR peaks associated to the Michael product

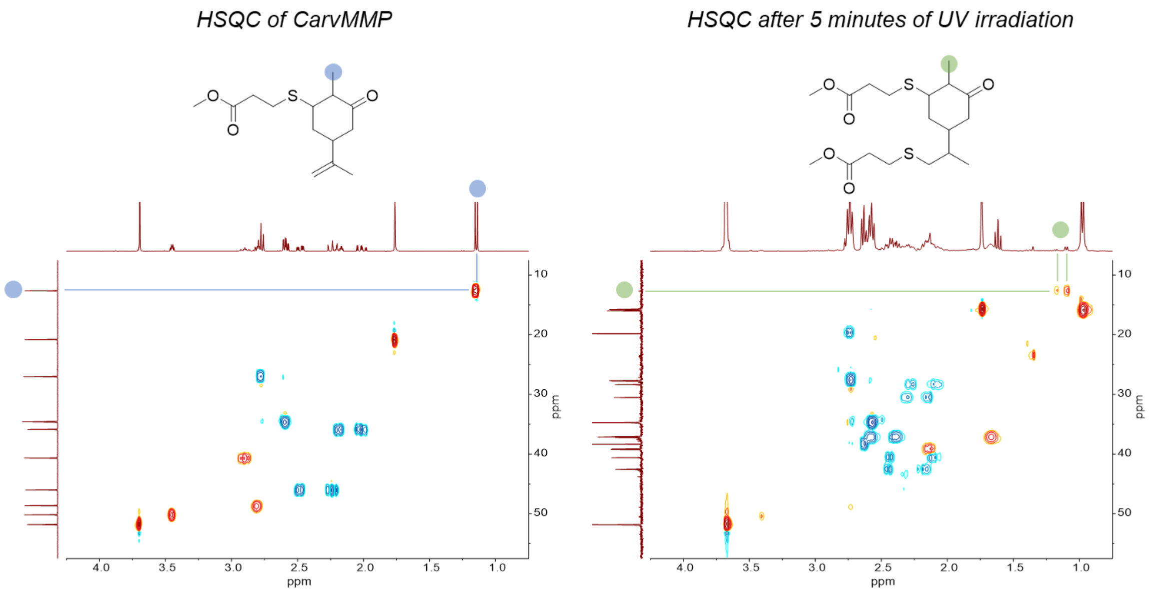

Figure S3. HSQC NMR spectra of CarvMMP (left) and crude thiol-ene kinetics experiment after 5 minutes of UV irradiation (right) in $CDCl_3$.

Identifying peaks in the ^{1}H NMR that corresponded to the desired Michael product relied on ^{13}C NMR due to the presence of stereoisomers. The HSQC of the assigned CarvMMP identified the ^{13}C shift corresponding to the α-methyl ketone of the Michael product. A further HSQC of the thiol-ene kinetics experiment taken after 5 minutes of irradiation highlights the corresponding ^{1}H peaks of the Michael product that can be integrated against the consumption of the enone. Full assignment of CarvMMP in $CDCl_3$ can be found below.

Dynamic exchange experiment at ambient temperature

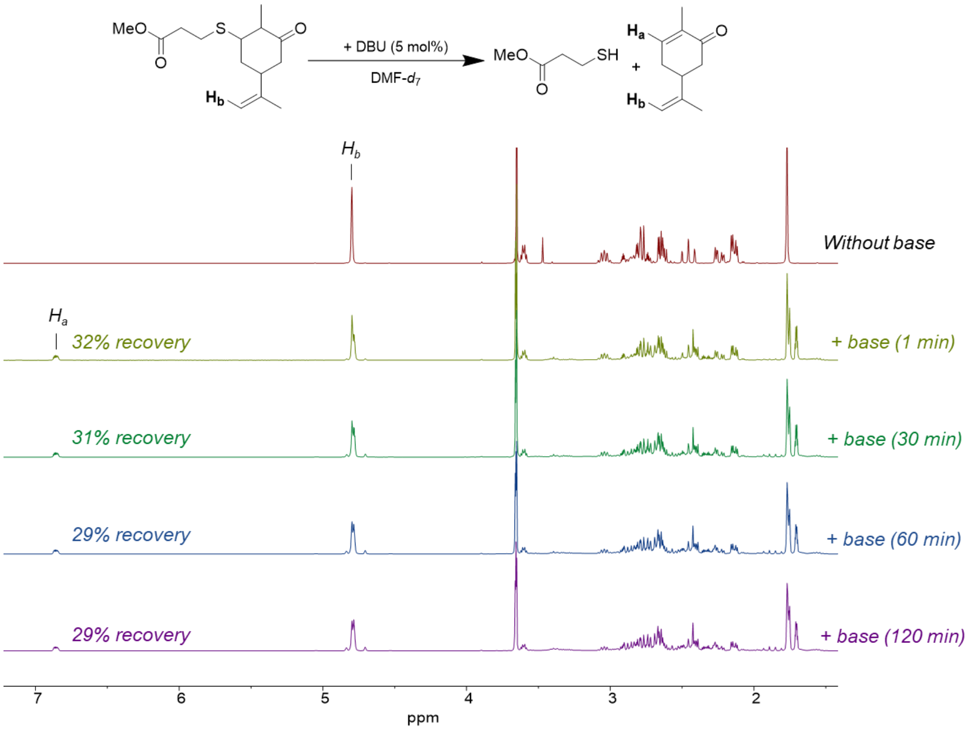

Figure S4. The dynamic exchange of CarvMMP at ambient temperature with and without DBU (5 mol%) at different reaction times. Enone peak at 6.85 ppm was quantified from the unconsumed isopropenyl singlet at 4.80 ppm in DMF-d_7.

Van’t Hoff plot

Calculation of the K_{eq} for the dissociation of CarvMMP in DMF-d_7 with DBU (5 mol%) was achieved from quantifying the carvone concentration at increasing reaction temperatures. Quantifying the L-carvone concentration was achieved through the integration of the characteristic enone singlet at 6.85 ppm in DMF-d_7 against the propenyl singlet at 4.80 ppm in DMF-d_7 which was unconsumed in the process. Assuming the absence of side reactions during the experiment, the concentration of Carvone and MMP will be equal. The concentration of CarvMMP can also be obtained from the known initial concentration and the concentration of the dissociated species.

$$K_{eq} = \frac{[Carvone][MMP]}{[CarvMMP]}$$

Equation S3. Equation for K_{eq} of the dissociation of CarvMMP.

$$K_{eq} = \frac{[Carvone]^2}{([CarvMMP_{int}] - [Carvone])}$$

Equation S4. Equation for K_{eq} using the carvone and initial CarvMMP concentration.

Temperature (°C)	1000/T (K^{-1})	K_{eq}	ln(K_{eq})
50	3.096	0.471	-0.753
60	3.003	0.672	-0.397
70	2.915	1.281	0.248
80	2.833	2.106	0.745
90	2.755	3.453	1.239
100	2.681	5.283	1.664

Table S2. Data summary used to construct the Van’t Hoff plot.

Photorheology 1-Carv$_{Resin}$ and a L-carvone and 3-arm thiol mixture

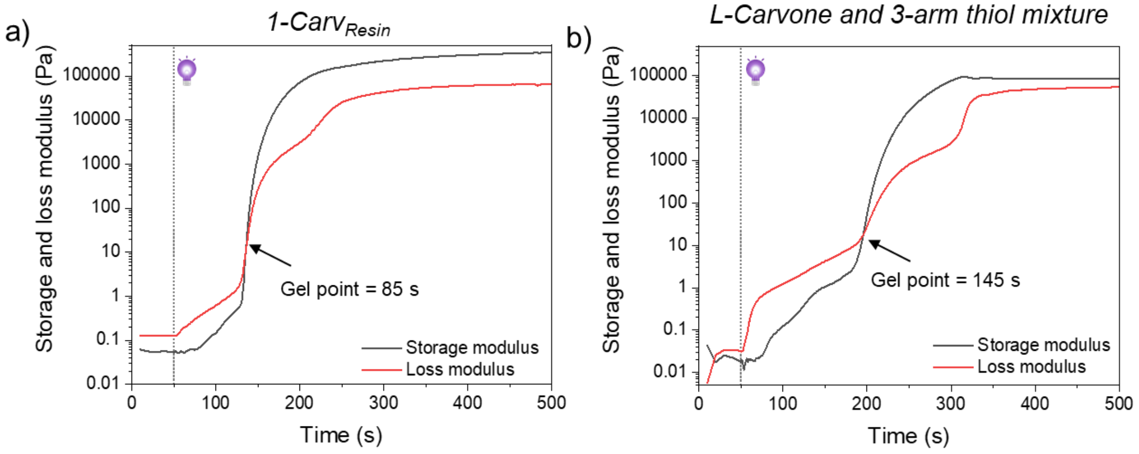

Figure S5. Photorheological comparison of a prepolymer system (1-Carv$_{Resin}$) and a non-prepolymer control.

Viscosity of resin systems

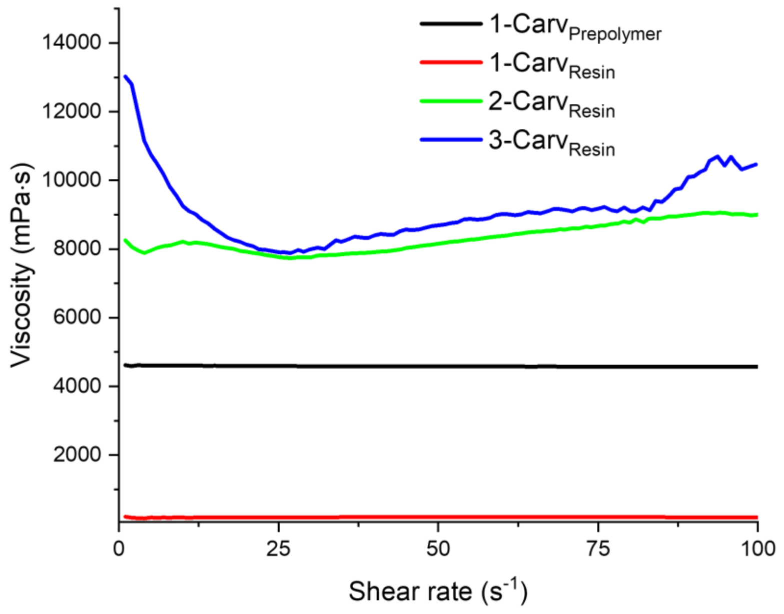

Figure S6. Viscosity between a shear rate of 1 to 100 s^{-1} for 1-Carv$_{Prepolymer}$, 1-Carv$_{Resin}$, 2-Carv$_{Resin}$ and 3-Carv$_{Resin}$.

Improved weight recovery through precipitation into distilled water

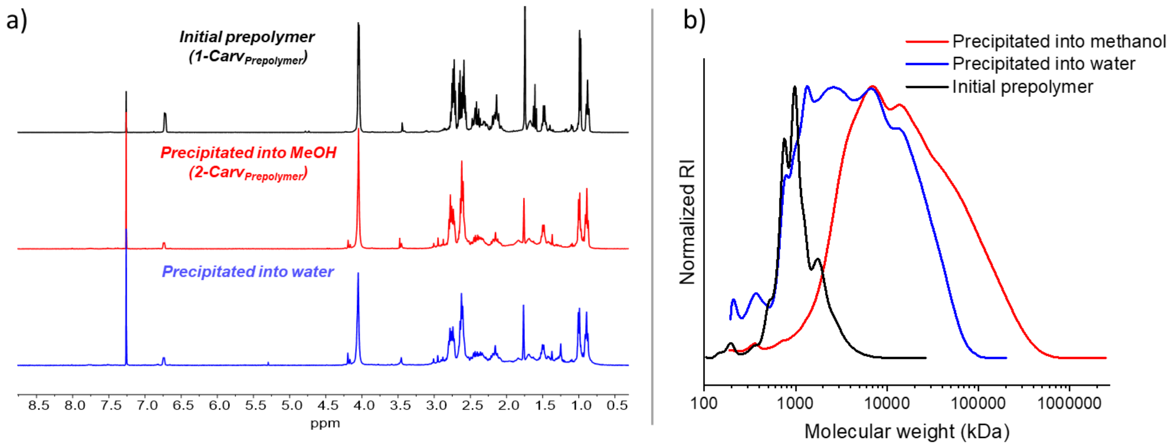

Figure S7. a) ^{1}H NMR spectra of the initial prepolymer (1-Carv$_{Prepolymer}$), the methanol precipitated (2-Carv$_{Prepolymer}$) and the depolymerization attempt precipitated into distilled water in $CDCl_3$, 400 MHz. b) SEC elugrams of the initial prepolymer (1-Carv$_{Prepolymer}$), the methanol precipitated (2-Carv$_{Prepolymer}$) and the depolymerization attempt precipitated into distilled water ($CHCl_3$, v/v 0.5% NEt_3, against polystyrene standards).

We hypothesized the material weight recovery was non-quantitative due to the loss of low molecular weight fractions during the precipitation step. Using distilled water (as opposed to methanol) afforded a higher material recovery of 71% (55% for methanol precipitation). The ^{1}H NMR spectra were alike, with both methods regenerating the desired enone species. The SEC elugrams indicate that lower molecular weight species are recovered when precipitating into distilled water, which supports our initial hypothesis that non-quantitative mass recovery was a result of the lower molecular weight species being solubilized during precipitation into methanol.

UV-Vis of prepolymers

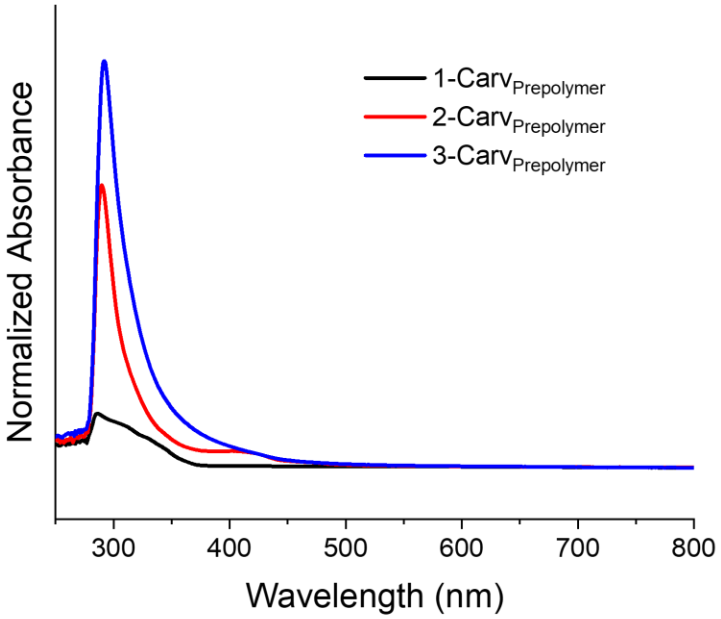

Figure S8. Normalized absorbance of all prepolymers (1-Carv$_{Film}$, 2-Carv$_{Film}$ and 3-Carv$_{Film}$) between 250 – 800 nm.

Tensile data of the post-cured films

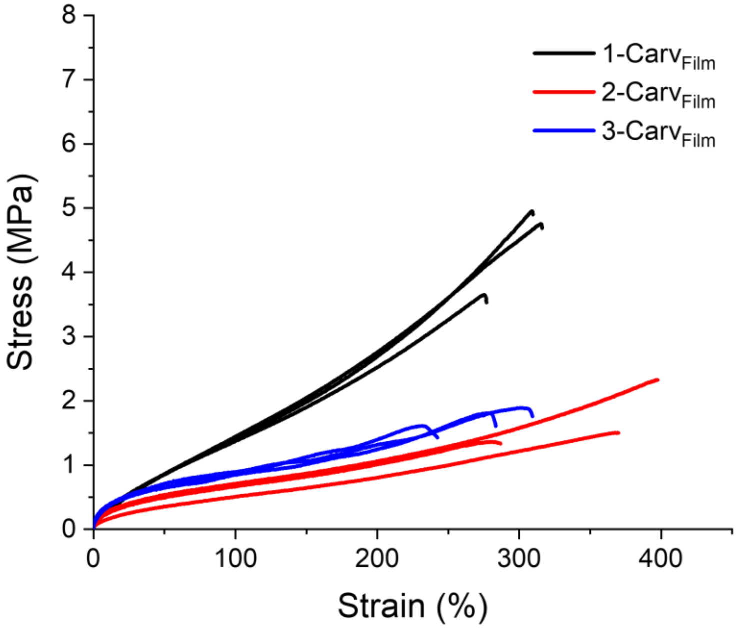

Figure S9 Stress vs strain tensile curves of post-cured films, 1-Carv$_{Film}$, 2-Carv$_{Film}$ and 3-Carv$_{Film}$. (n=3)

Table S3. Tensile data for the post-cured films 1-Carv$_{FIlm}$, 2-Carv$_{Film}$ and 3-Carv$_{Film}$. (n=3)

	E (MPa)	**Stress at break** (MPa)	**Strain at Break** (%)	**U_T** (MJm^{-3})
1-Carv$_{Film}$	4.1 ± 0.3	4.4 ± 0.8	300 ± 21	6.4 ± 1.2
2-Carv$_{Film}$	3.8 ± 1.0	1.7 ± 0.5	351 ± 57	3.3 ± 1.1
3-Carv$_{Film}$	6.2 ± 0.6	1.8 ± 0.2	273 ± 37	2.9 ± 0.6

Comparison of ^{1}H NMR spectra of the depolymerized networks

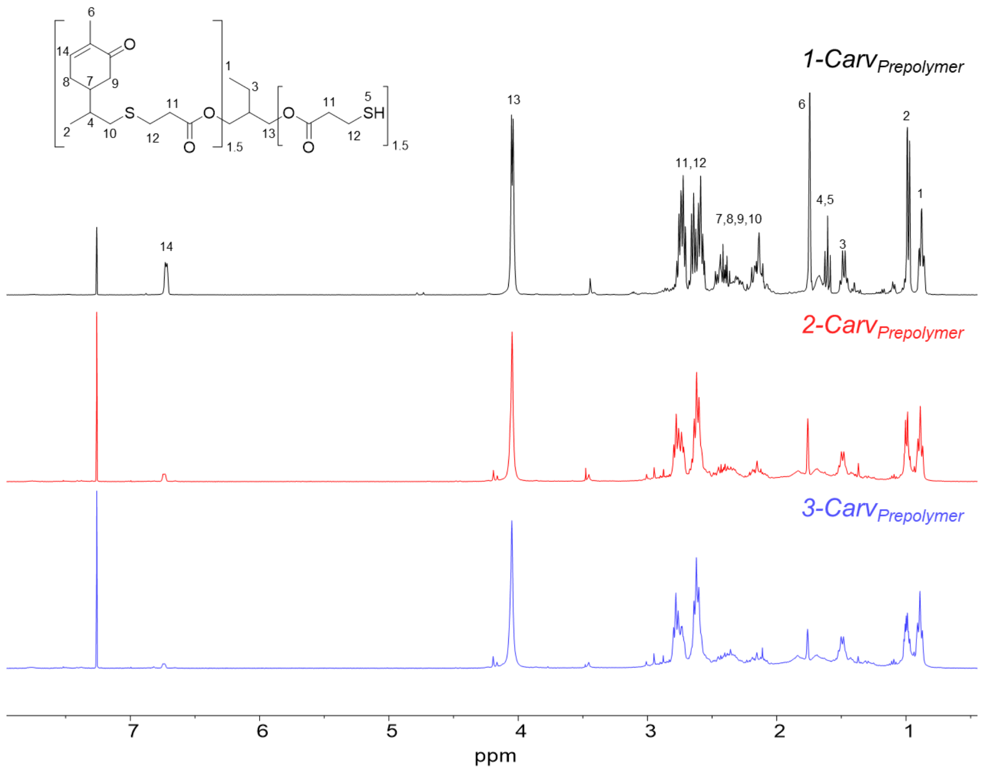

Figure S10. ^{1}H NMR spectra of the 1-Carv$_{Prepolymer}$, 2-Carv$_{Prepolymer}$ and 3-Carv$_{Prepolymer}$ in $CDCl_3$, 400 MHz.

NMR Data

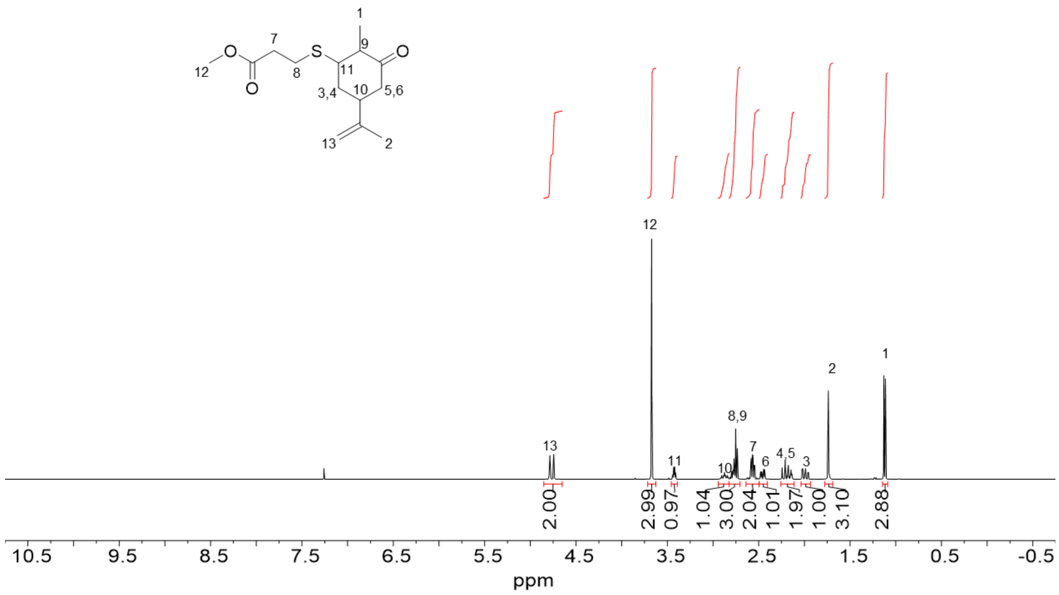

Figure S11. CarvMMP ^{1}H NMR Spectrum – 400 MHz, $CDCl_3$.

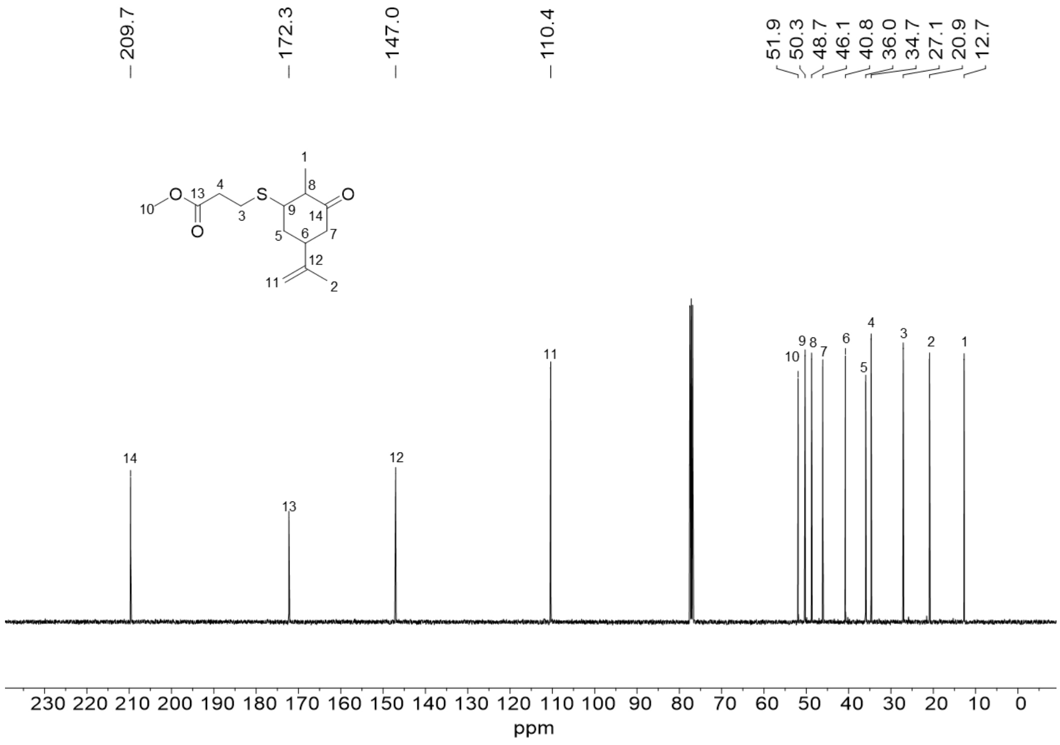

Figure S12. CarvMMP ^{13}C NMR Spectrum – 101 MHz, $CDCl_3$.

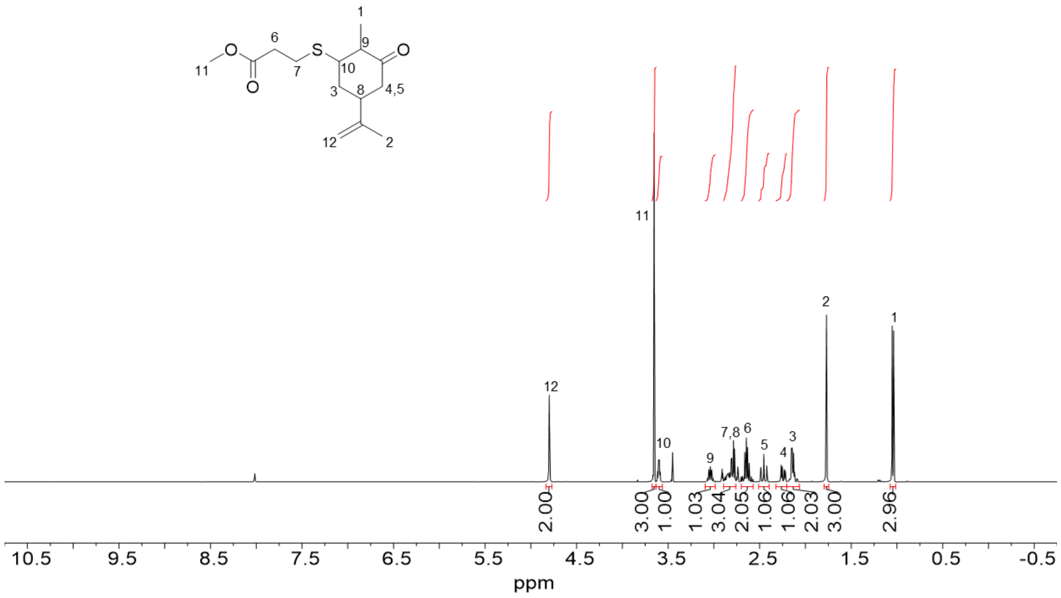

Figure S13. CarvMMP ¹H NMR Spectrum – 400 MHz, DMF-d_7.

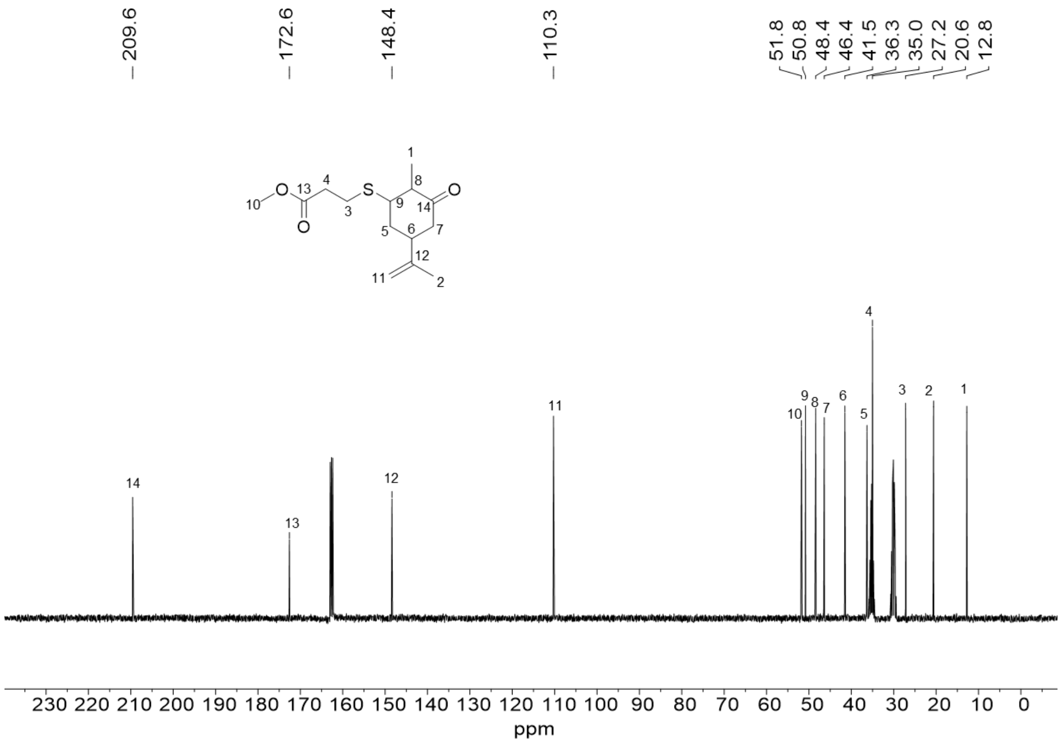

Figure S14. CarvMMP ¹³C NMR Spectrum – 101 MHz, DMF-d_7.

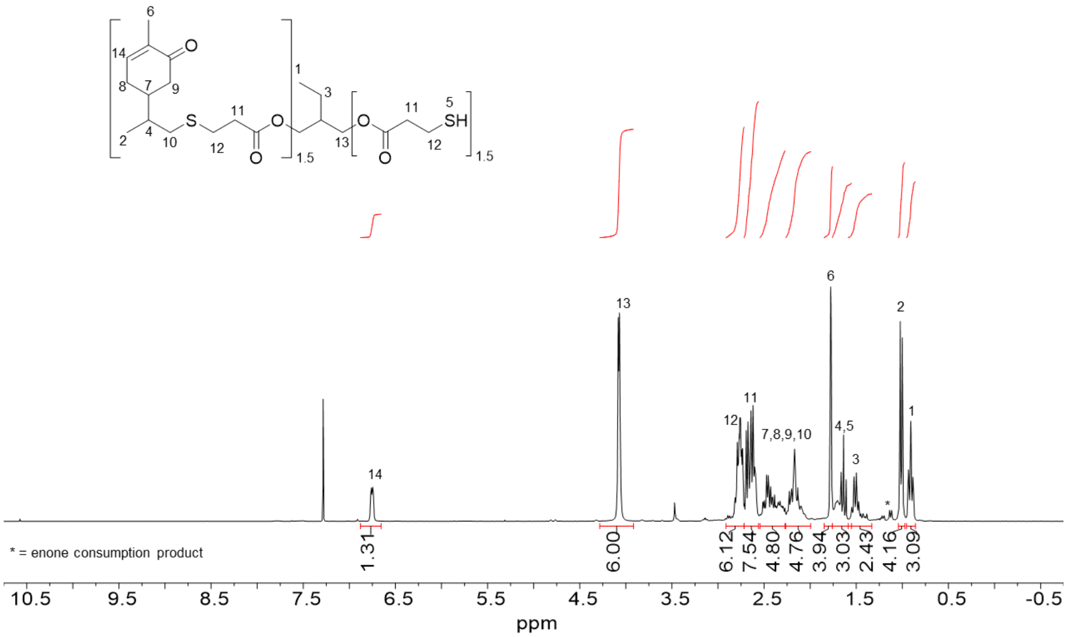

Figure S15. 1-Carv$_{Prepolymer}$ ^{1}H NMR Spectrum – 400 MHz, $CDCl_3$.

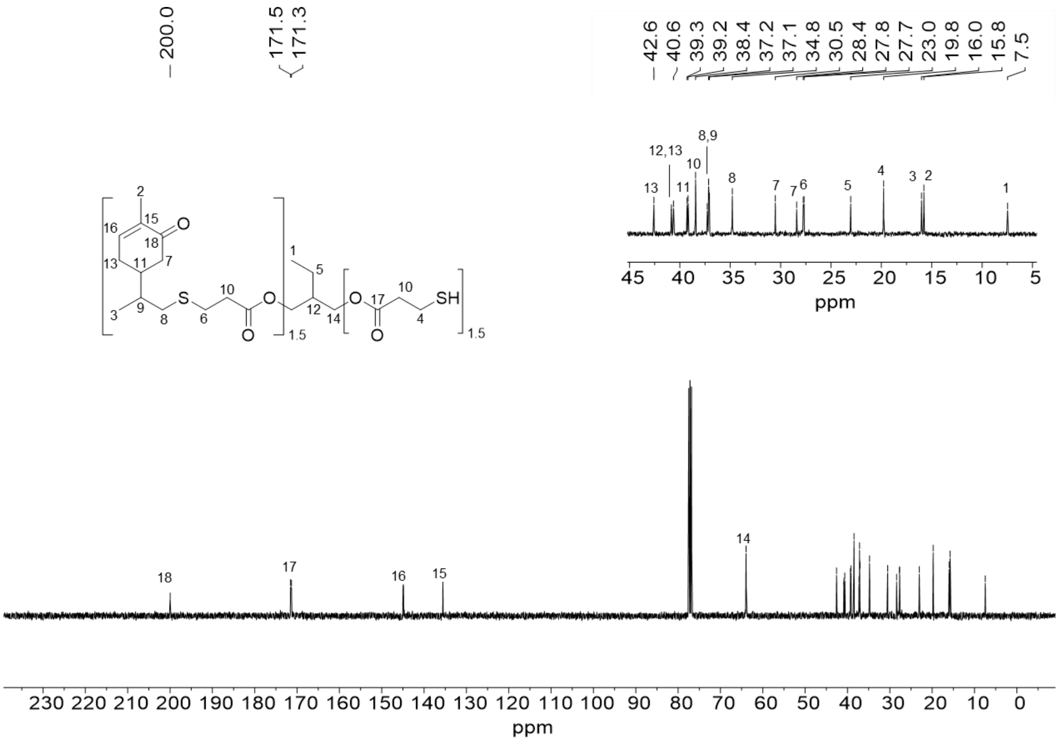

Figure S16. 1-Carv$_{Prepolymer}$ ^{13}C NMR Spectrum – 101 MHz, $CDCl_3$.

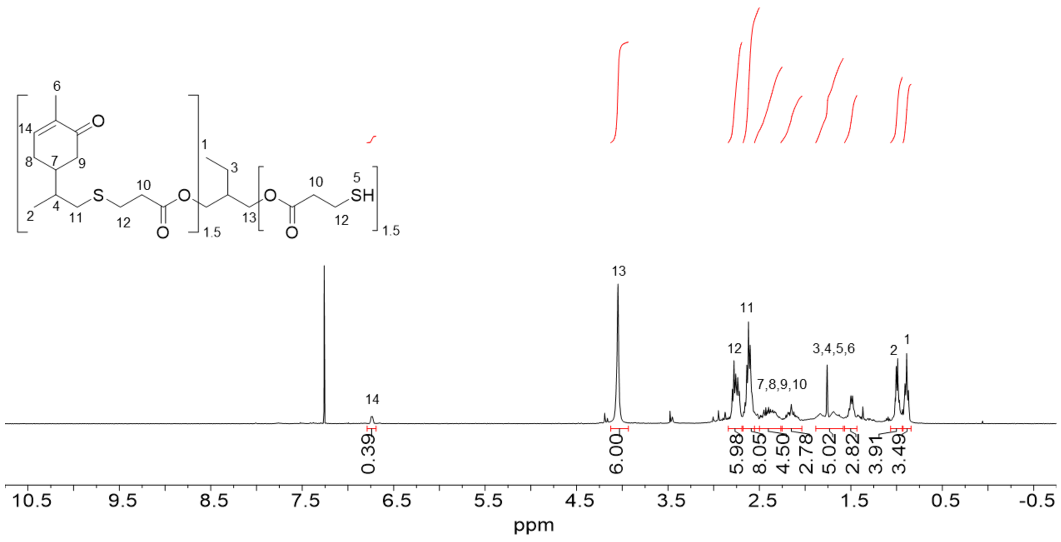

Figure S17. 2-Carv$_{Prepolymer}$ ^{1}H NMR Spectrum – 400 MHz, $CDCl_3$.

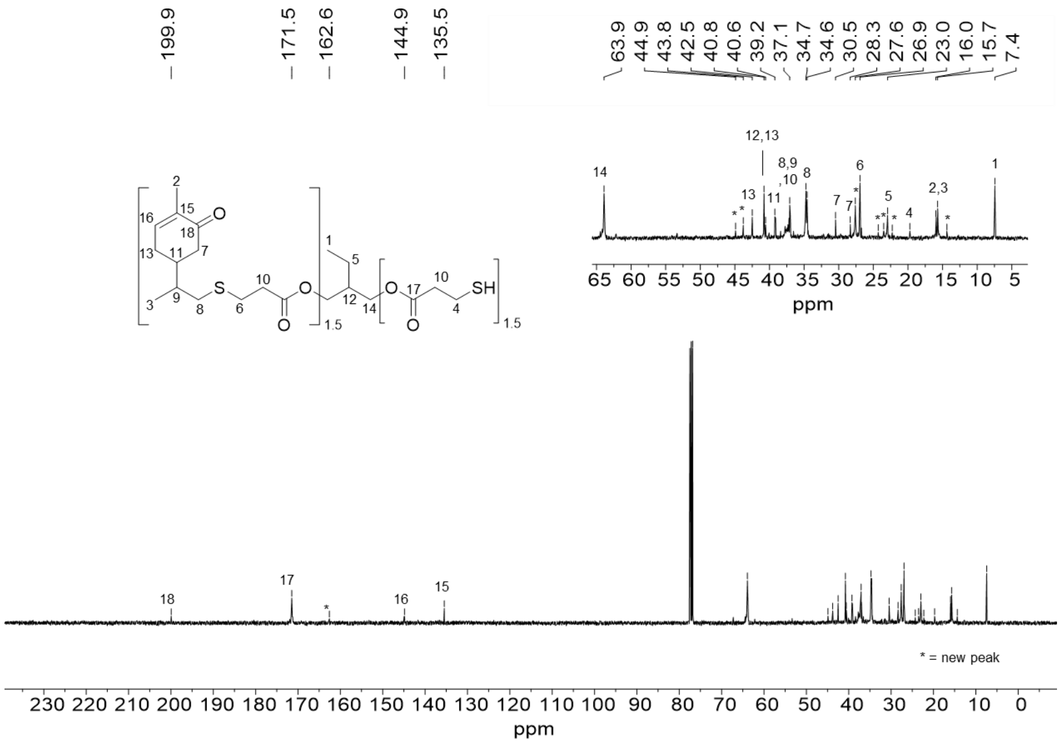

Figure S18. 2-Carv$_{Prepolymer}$ ^{13}C NMR Spectrum – 101 MHz, $CDCl_3$.

Differential Scanning Calorimetry thermograms

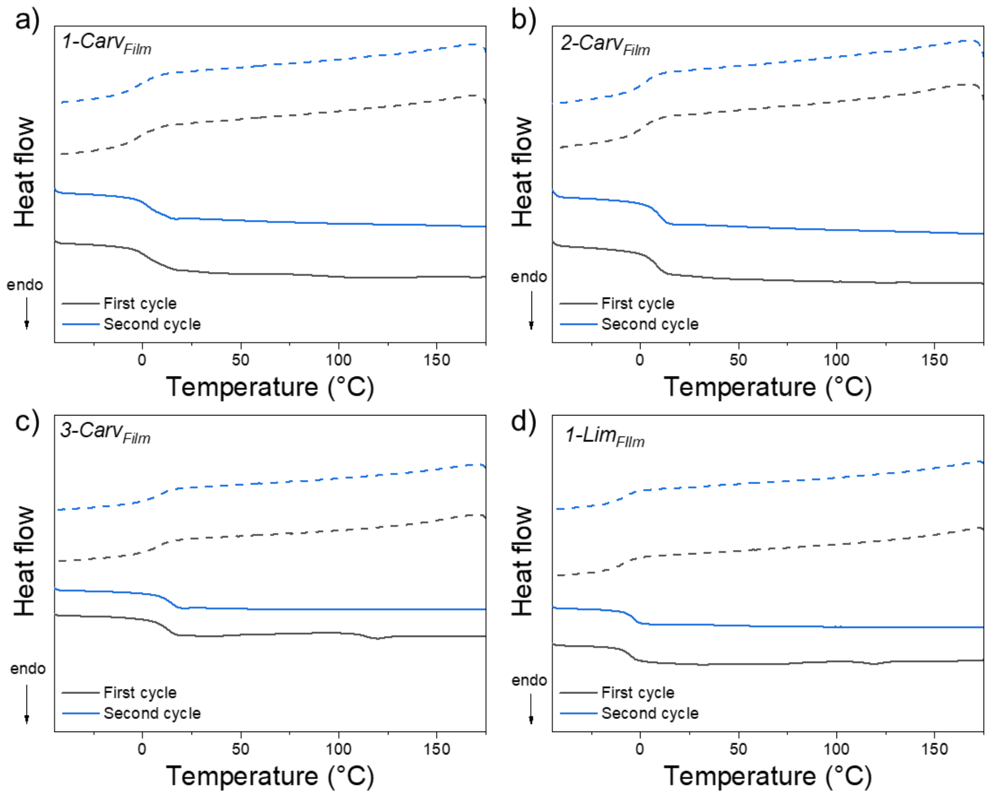

Figure S19. DSC thermograms of a) 1-Carv$_{Film}$, b) 2-Carv$_{Film}$, c) 3-Carv$_{Film}$, d) 1-Lim$_{Film}$.

Thermogravimetric Analysis Data

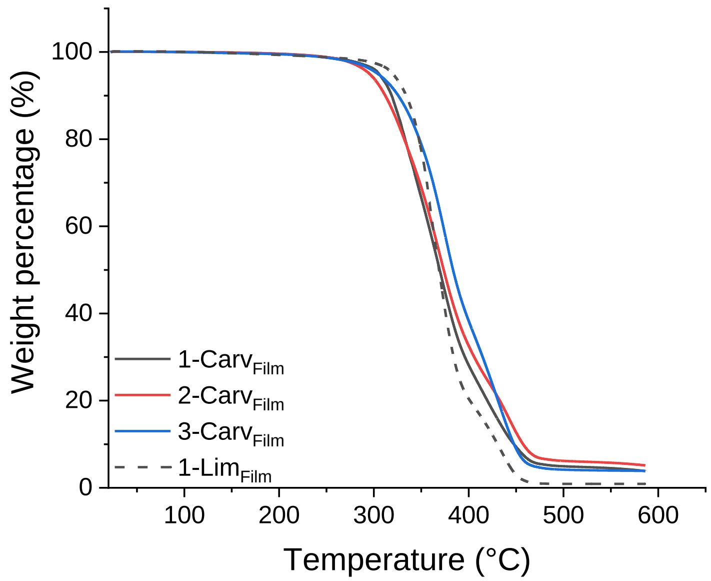

Figure S20. TGA thermograms of L-carvone and limonene networks.

Fourier Transform Infrared spectra of prepolymers and carvone networks

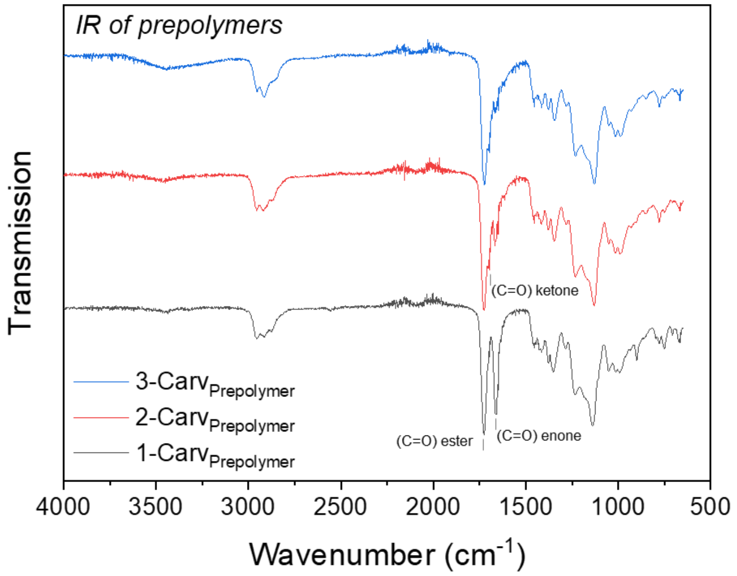

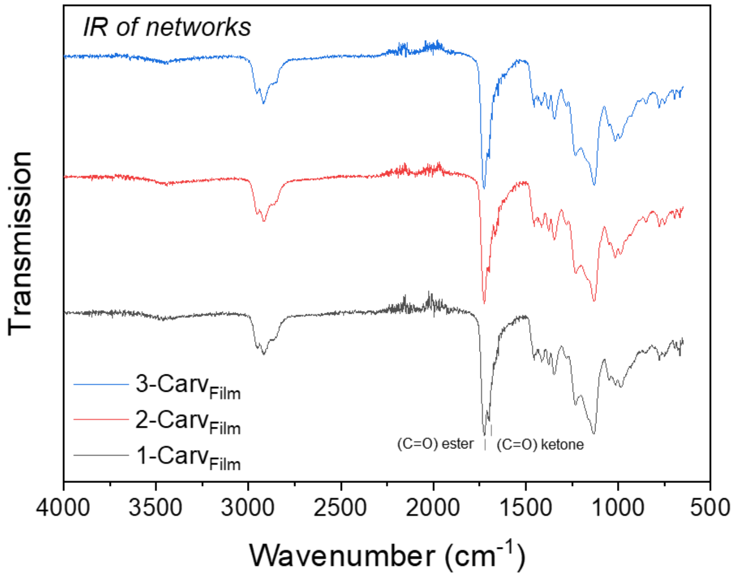

Figure S21. FT-IR spectra of the prepolymers (top) and the post-cured network (bottom).

Photorheology

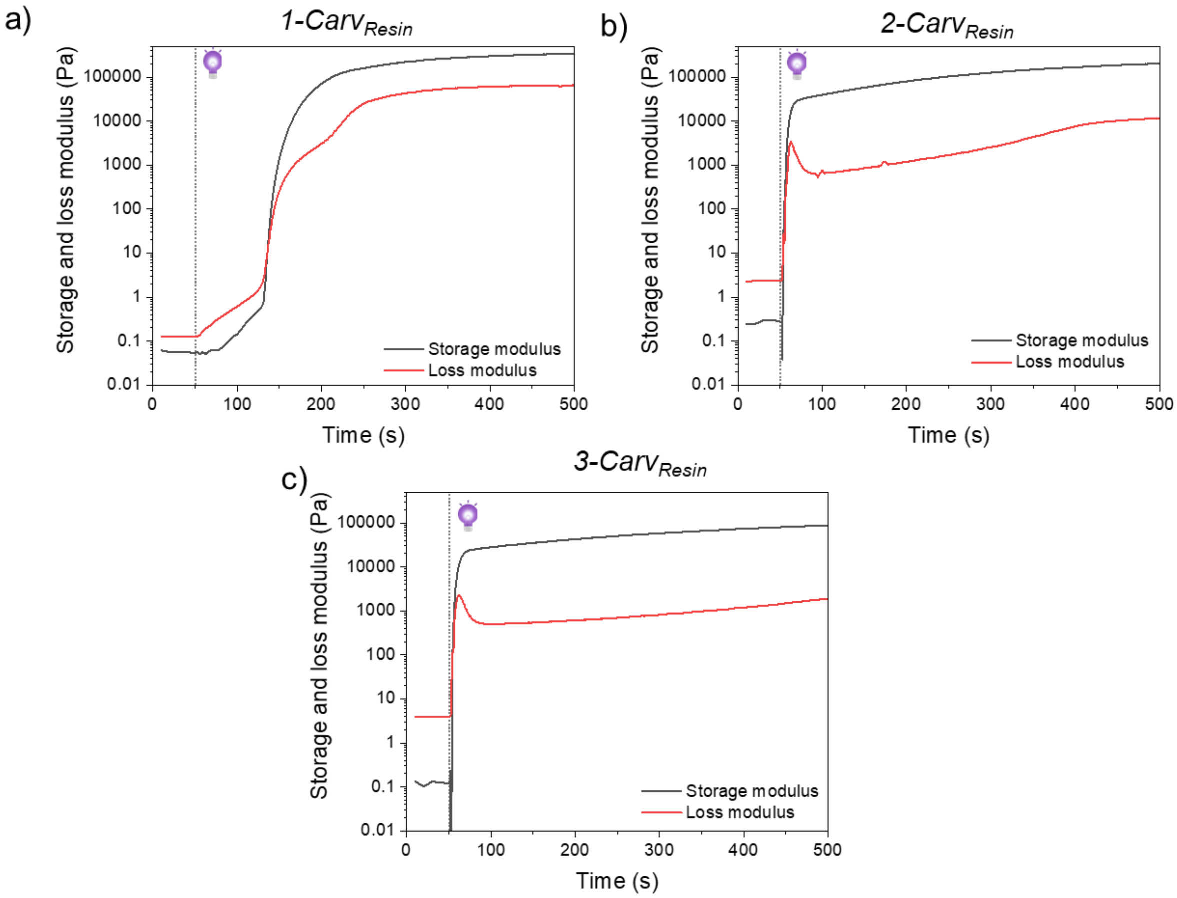

Figure S22. Storage and loss modulus profiles over 500s obtained from photorheology for a) 1-Carv$_{Film}$, b) 2-Carv$_{Film}$, c) 3-Carv$_{Film}$.

Dynamic Mechanical Analysis Thermograms

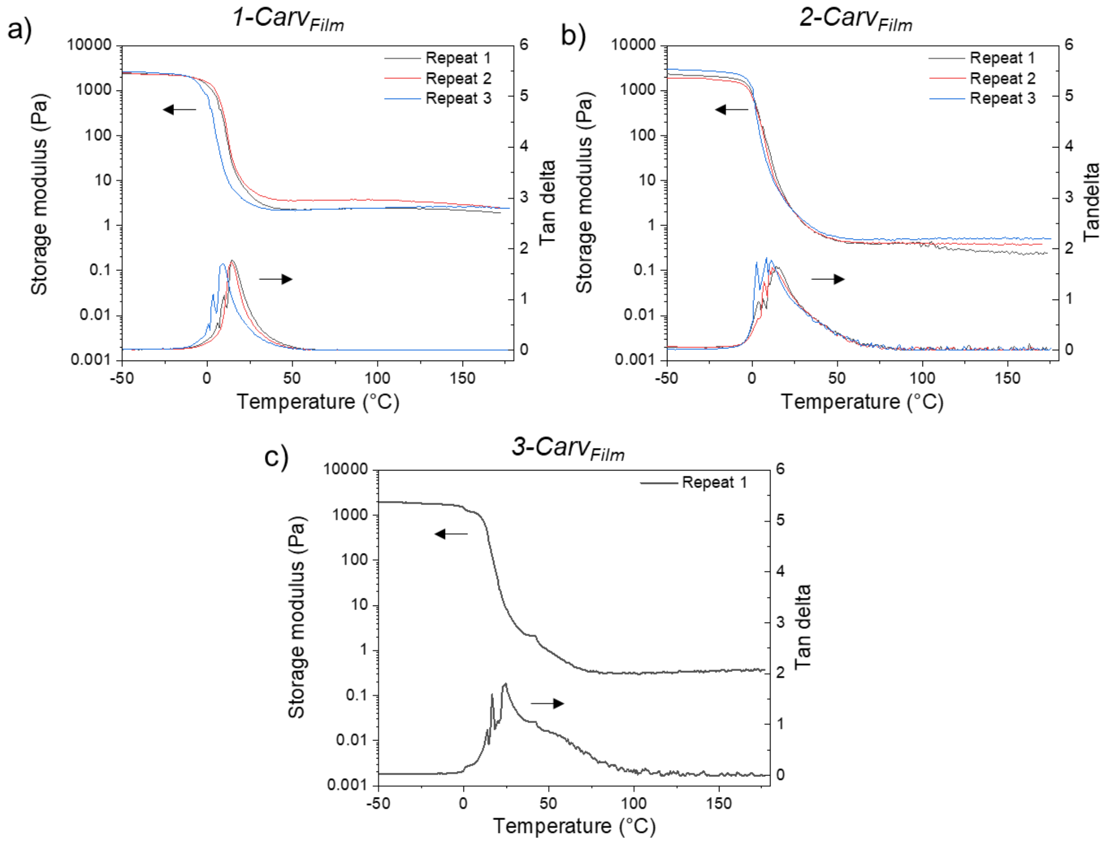

Figure S23. DMTA thermograms of a) 1-Carv$_{Film}$, b) 2-Carv$_{Film}$, c) 3-Carv$_{Film}$.

Stress relaxation experiments

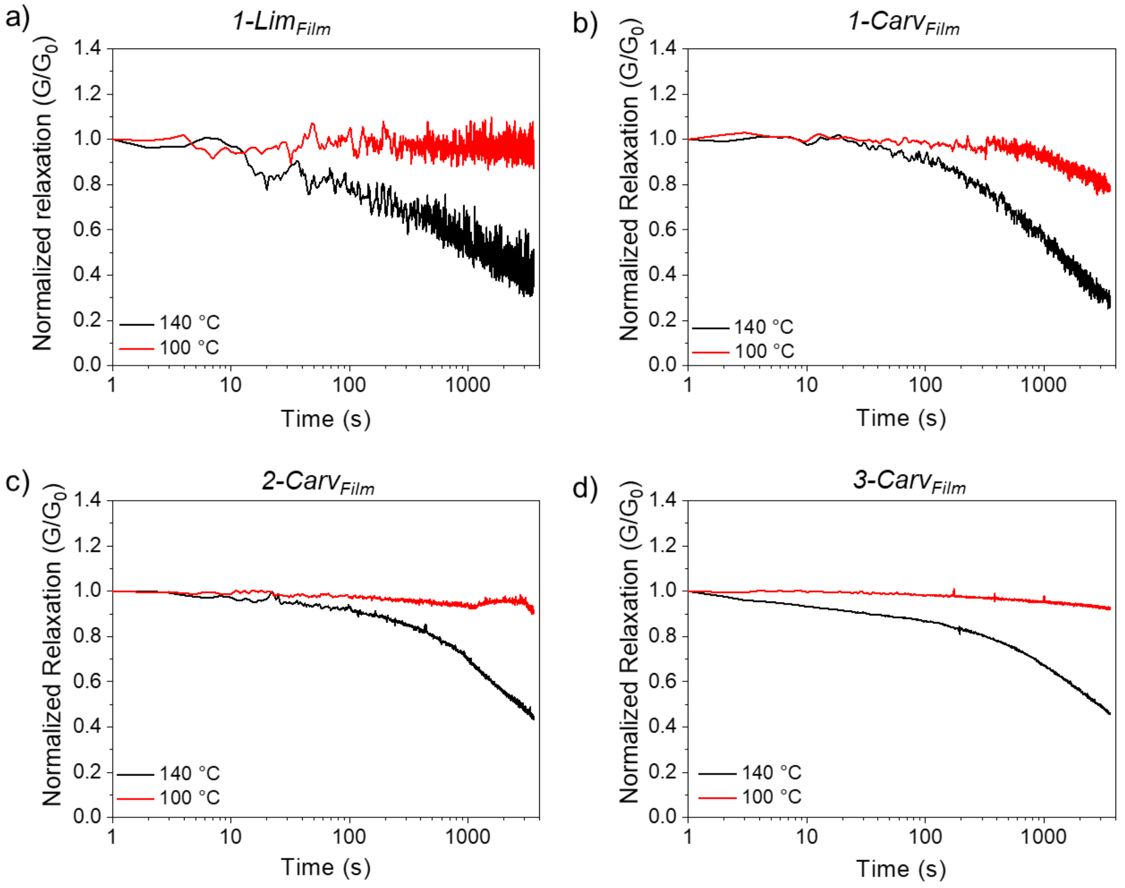

Figure S24. Stress relaxation experiments conducted at 140 and 100 °C for a) 1-Lim$_{Film}$, b) 1-Carv$_{Film}$, c) 2-Carv$_{Film}$, d) 3-Carv$_{Film}$.

Thermomechanical properties summary

Table S4. Summary of thermal and mechanical properties for the cured carvone and limonene network.

**Network**	T_g **1st cycle** (°C)[a]	T_g **2nd cycle** (°C)[a]	**Tan delta peak** (°C)[b]	**Gel fraction** (%)	**Degree of swelling** (%)	$T_{d,5\%}$ (°C)[c]	**Storage modulus @ -25 °C** (Pa)[d]	**Storage modulus @ 100 °C** (Pa)[d]
**1-Carv$_{Film}$**	1	8	12 ± 3	89.4 ± 0.3	239.2 ± 2.9	305	2292 ± 104	2.87 ± 0.74
**2-Carv$_{Film}$**	8	10	12 ± 3	80.6 ± 2.1	315 ± 38.7	295	2202 ± 483	0.43 ± 0.07
**3-Carv$_{Film}$**	13	15	24[§]	85.4 ± 2.7	357.4 ± 2.3	304	1823	0.3
**1-Lim$_{Film}$**	-5	-3	-	99.5 ± 0.3	261.1 ± 8.3	319	-	-

[a] T_g calculated from DSC thermograms [b] Obtained from DMA thermograms (*n=3*) [c] Temperature at 5% weight loss obtained from TGA thermograms [d] Storage modulus obtained from DMA at specified temperature. [§] (*n=1*)

Resin summary

Table S5. Summary of the carvone resins.

Resin	M_n (kDa)[a]	M_w (kDa)[a]	$Đ$[a]	**Enone content** (%)[b]	**Gelation time** (s)[c]	**Viscosity** (Pa·s)[d]	**Storage modulus @ 400s** (kPa)[e]
**1-Carv$_{Resin}$**	0.9	1.2	1.4	94	85	0.2	297
**2-Carv$_{Resin}$**	5.8	36.1	6.3	31	4	8.3	167
**3-Carv$_{Resin}$**	6.0	31.1	5.2	19	4	8.9	73

[a] Obtained from GPC against polystyrene standards in chloroform. [b] Calculated from ^{1}H-NMR spectroscopy [c] Obtained from the intersection of storage and loss modulus in photorheology [d] Obtained from a rheological shear rate sweep [e] Storage modulus from photorheology after 400s of irradiation.
